# Pharmacological Modulation of Glutamatergic and Neuroinflammatory Pathways in a Lafora Disease Mouse Model

**DOI:** 10.1007/s12035-022-02956-7

**Published:** 2022-07-14

**Authors:** Belén Mollá, Miguel Heredia, Ángela Campos, Pascual Sanz

**Affiliations:** 1 Laboratory of Nutrient Signaling, Institute of Biomedicine of Valencia (CSIC), Valencia, Spain; 2 Centro de Investigación Biomédica en Red de Enfermedades Raras (CIBERER), 46010 Valencia, Spain; 3 Instituto de Biomedicina de Valencia, Consejo Superior de Investigaciones Científicas, Jaime Roig 11, 46010 Valencia, Spain

**Keywords:** Lafora disease, Cognitive impairment, Neuroinflammation, Riluzole, Memantine, Resveratrol, Minocycline

## Abstract

Lafora disease (LD) is a fatal rare neurodegenerative disorder that affects young adolescents and has no treatment yet. The hallmark of LD is the presence of polyglucosan inclusions (PGs), called Lafora bodies (LBs), in the brain and peripheral tissues. LD is caused by mutations in either *EPM2A* or *EPM2B* genes, which, respectively, encode laforin, a glucan phosphatase, and malin, an E3-ubiquitin ligase, with identical clinical features. LD knockout mouse models (*Epm2a − / − *and *Epm2b − / −*) recapitulate PG body accumulation, as in the human pathology, and display alterations in glutamatergic transmission and neuroinflammatory pathways in the brain. In this work, we show the results of four pre-clinical trials based on the modulation of glutamatergic transmission (riluzole and memantine) and anti-neuroinflammatory interventions (resveratrol and minocycline) as therapeutical strategies in an *Epm2b − / − *mouse model. Drugs were administered in mice from 3 to 5 months of age, corresponding to early stage of the disease, and we evaluated the beneficial effect of the drugs by in vivo behavioral phenotyping and ex vivo histopathological brain analyses. The behavioral assessment was based on a battery of anxiety, cognitive, and neurodegenerative tests and the histopathological analyses included a panel of markers regarding PG accumulation, astrogliosis, and microgliosis. Overall, the outcome of ameliorating the excessive glutamatergic neurotransmission present in *Epm2b − / − *mice by memantine displayed therapeutic effectiveness at the behavioral levels. Modulation of neuroinflammation by resveratrol and minocycline also showed beneficial effects at the behavioral level. Therefore, our study suggests that both therapeutical strategies could be beneficial for the treatment of LD patients.

**Graphical abstract:**

A mouse model of Lafora disease (*Epm2b-/-*) was used to check the putative beneficial effect of different drugs aimed to ameliorate the alterations in glutamatergic transmission and/or neuroinflammation present in the model. Drugs in blue gave a more positive outcome than the rest.

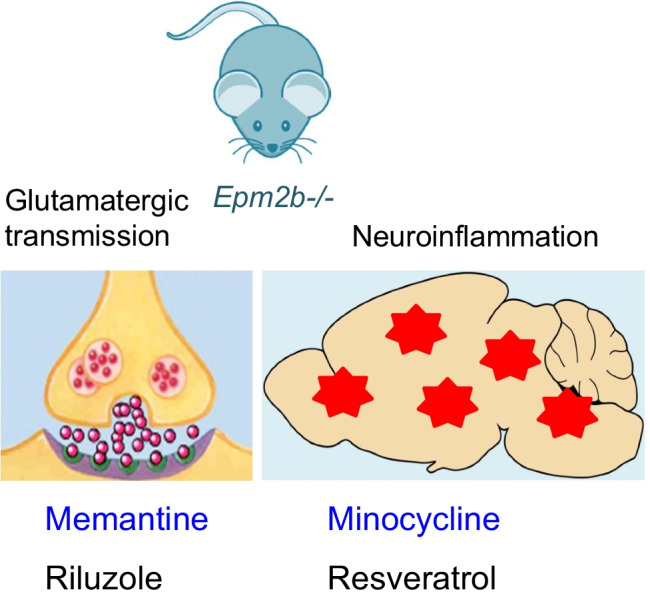

**Supplementary Information:**

The online version contains supplementary material available at 10.1007/s12035-022-02956-7.

## Introduction

Lafora disease (LD; OMIM#254780) is an inherited uncommon juvenile neurodegenerative illness that proceeds through persistent epileptic seizures, cognitive impairment, and fast neurological deterioration to mortality within 10 years from onset [1]. The buildup of insoluble polyglucosan inclusions (PGs), known as Lafora bodies (LBs), primarily in the brain, is an early characteristic of LD [2, 3], contributing to symptomatology and remains unalterable throughout the illness. There is currently no effective therapy available, neither restorative nor palliative, to slow the course or alleviate any of the symptoms of LD [4]. In this context, the International Lafora Epilepsy Cure Initiative (LECI) has made a significant joint effort to identify an LD therapeutic or cure [5].

Mutations in either *EPM2A* or *EPM2B/NHLRC1* genes, which, respectively, encode laforin, a glucan phosphatase, and malin, an E3-ubiquitin ligase, have been described in LD patients [6–8]. Laforin and malin assemble into a functional complex involved in glycogen metabolism, as part of a quality control mechanism to prevent the accumulation of insoluble glycogen [9, 10]. LD knockout (KO) mouse models with a complete loss of function of laforin (*Epm2a − / −*) [11] or malin (*Epm2b − / −*) [12–14] have been used to understand LD pathophysiology. Although they do not fully reproduce human pathophysiology since they do not present spontaneous seizures and they do not die as the human patients do, they partially mimic human symptoms such as early PG accumulation in muscle, heart, and brain from 2 months of age [11, 14]; they also show slight impairment of motor coordination, abnormal postures of the hindlimb, memory defects, and spontaneous myoclonic seizures evident from 9 months of age [11, 14, 15]; finally, they present enhanced excitability [11] with hyperactive behavior from 1 to 11 months old [16, 17].

At the histopathological level, the brain of LD mouse models shows neurodegeneration with loss of GABAergic and parvalbumin + (PV +) neurons and dendritic abnormalities in pyramidal neurons [16–18], massive astrogliosis accumulating PGs [19, 20], as well as an early neuroinflammatory status from 3 months of age [21, 22]. Due to the scarcity of accessing to human brain samples from Lafora disease patients, only the accumulation of insoluble polyglucosan inclusions (PGs) has been confirmed in the LD human brains [2, 3].

Since astrocytic functionality seems to be compromised in LD mouse models, our laboratory has studied the possible alterations in excitatory glutamatergic transmission to gain insight into the glutamatergic system, as an essential regulator of epileptic seizures. Successfully, Muñoz-Ballester et al. [23] unravelled a decreased level of glutamate transporter GLT-1 at the plasma membrane of LD astrocytes, which might underlie the in vivo glutamate clearance defects present in LD mouse models [24]. Furthermore, Perez-Jimenez et al. [25] confirmed that such defective alterations are related to insufficient ubiquitination of GLT-1 due to the absence of a functional laforin/malin complex. On the basis of these strong data, it became clear that an altered excitatory glutamatergic system might be behind the sensitivity to convulsant agents observed in LD mouse models [26, 27]. Recently, our laboratory has also been involved in defining neuroinflammation as a novel hallmark of LD. In this regard, we have described progressive neuroinflammation in the brain of LD mice which increasingly worsens from 3 to 16 months of age [22].

The work we present in this manuscript is an extension of the previous achievements of our laboratory in preclinical studies in LD by using metformin [28], which led to the designation of metformin as an orphan drug for the treatment of Lafora disease by the European Medicines Agency (EMA) and the US Food and Drug Administration (FDA). In addition, we have recently reported the beneficial effect of the administration of propranolol, not only in memory and attention defects but also in the accumulation of polyglucosan inclusions, neuronal disorganization, astrogliosis, and microgliosis in the hippocampus of LD mice [29]. Following this line of work, and to repurpose further drugs for LD, in this work we have carried out four novel pre-clinical studies in *Epm2b − / − *mice with two different strategies of intervention based on targeting glutamatergic transmission (riluzole and memantine) as well as neuroinflammation (resveratrol and minocycline).

Riluzole is the first FDA-approved medication for amyotrophic lateral sclerosis (ALS) due to its capability to modulate glutamate neurotransmission by inhibiting both presynaptic glutamate release and postsynaptic glutamate receptor signaling [30]. Riluzole has been effective in delaying median time to death in a mouse model of ALS [31] and cognitive decline in Alzheimer’s disease (AD) mouse models [32, 33]. On the other hand, memantine is an antagonist of postsynaptic glutamate NMDA receptors, which reduces the effects of excitotoxic glutamate release. This compound is regularly prescribed to improve the cognitive impairments in AD [34, 35].

Resveratrol is known worldwide as a nutraceutical for its anti-oxidative, anti-aging, and anti-inflammatory properties [36, 37], but it also has been recognized as a promising therapeutic agent against chronic neuroinflammation and neurodegeneration in AD [38, 39]. In the same light, minocycline, a classical tetracycline antibiotic, has also been reported with novel anti-inflammatory and neuroprotective activities in several neurodegenerative conditions such as ALS, AD, or Huntington’s disease [40, 41].

In this work, we have administered these compounds to 3-month-old *Epm2b − / − *mice (corresponding to early stages of LD) for 2 months. In these mice, we have analyzed neurodegenerative status and cognitive tasks (memory and anxiety-like behavior), as well as histopathological hallmarks in the brain [presence of polyglucosan inclusions (PGs), microgliosis, and astrogliosis] to evaluate the effectiveness of all pharmacological interventions. We found an improvement in the performance of behavioral tests and an amelioration of neurodegenerative signs by modulating neuroinflammation with resveratrol and minocycline and also by promoting neuroprotection with memantine. In the case of riluzole, we did not find any significant beneficial effect. Therefore, the results presented in this work support the potential beneficial effects of both interventional strategies in LD.

## Material and Methods

### Animals

Malin knockout mice (*Epm2b − / −*) on a pure C57BL/6JRccHsd background and the corresponding control mice (WT) [29] were maintained at the IBV-CSIC facility on a 12 light/dark cycle with food and water ad libitum. A total of 63 WT and 93 *Epm2b − / − *male mice were randomly assigned to untreated or treated groups along with four different pre-clinical trials (riluzole, memantine, resveratrol, and minocycline). The number of animals per group for each trial was as follows: 1) vehicle group (*N* = 7 WT and *N* = 6 *Epm2b − / − *mice) and treated group (*N* = 11 WT and *N* = 11 *Epm2b − / − *mice) for the riluzole study; 2) vehicle group (*N* = 15 WT and *N* = 15 *Epm2b − / − *mice) and treated group (*N* = 15 WT and *N* = 15 *Epm2b − / − *mice) for the resveratrol study; and 3) vehicle group (*N* = 15 WT and *N* = 15 *Epm2b − / − *mice) and treated group for memantine and minocycline study (*N* = 15 *Epm2b − / − *mice for each drug). The vehicle groups were administered with the corresponding vehicle used in the treated groups. Since no gender-link phenotype has been reported in mice or humans for Lafora disease, we used male mice to compare the results with previous data obtained in the lab.

### Drugs and Administration

All drugs tested in this work, riluzole (R116), memantine (M9292), and minocycline (M9511), were obtained from Sigma-Aldrich and trans-resveratrol (3,40,5-trihydroxy trans-stilbene, 70,675) from Cayman chemicals. Different pre-clinical trials were designed based on both the pathway (oral or intraperitoneal injection) and the drug solvent of the administration (water, saline, or vehicle). Thus, three pre-clinical trials were performed separately and consecutively: 1) the riluzole study was performed by oral administration in drinking water; 2) the resveratrol trial by intraperitoneal administration using vehicle solution (4% ethanol, 75 mM NaCl, 2.5% PEG4000, and 2.5% Tween20); and finally, 3) the memantine and minocycline assay by intraperitoneal administration in saline solution. Animals of 3 months of age either received riluzole (10 mg/kg/day) in drinking water or were injected intraperitoneally with vehicle solution alone or containing resveratrol (12 mg/kg), or with saline solution alone or containing memantine (25 mg/kg) or minocycline (25 mg/kg), in a volume of 100 µl, three times per week, during 2 months (Supplementary Fig. S1). Drug doses and administration schedules were based on a bibliographic search for all of them: riluzole [42, 43], resveratrol [44, 45], memantine [46], and minocycline [47]. These previous studies concluded that the doses we used in our assays were safe and that all compounds reached the brain to exert their effects.

### Behavioral Tests

After a 2-month treatment, animals were subjected to a battery of behavioral tests conducted during the light phase from 8:00 am to 3:00 pm. The order of the behavioral tests and resting time between them were the same for each mouse. The battery of behavioral tests consisted of five tests performed in the following order: hindlimb clasping, open field, elevated plus maze, Y-maze, and object location memory (OLM). Tests were conducted in order of increasing invasiveness: reflecting action, anxiety, and memory. Mice rested 48–72 h between tests (Supplementary Fig. S1). Behavioral tests were recorded by using the SMART Video Tracking software from PanLab/Harvard Apparatus to evaluate mouse movement. This advanced image analysis allows the recording of activity, trajectories, and a wide variety of standard calculations related to tracking such as time/distance/entries in zones both by user-defined zones and by the entire area of mazes. We used the following tests.

#### Hindlimb Clasping

Hindlimb clasping scores abnormal postures related to neurodegeneration and has been used as a marker of disease progression in a large number of neurodegenerative mouse models [48]. Mice were grasped by their tail for 10 s and hindlimb positions were scored from 0 to 3 [49]. If the hindlimbs were consistently splayed outward, away from the abdomen, it was assigned a score of 0 (absence). If one or two hindlimbs were partially retracted toward the abdomen for more than 5 s, it received a score of 1 (mild) or 2 (moderate), respectively. If both hindlimbs were completely retracted toward the abdomen, it received a score of 3 (severe).

#### Open Field Maze

The open field test is used to assess anxiety and exploratory behaviors [50]. Mice were placed in the middle of a peripheral zone of the arena (a wall-enclosed 50 cm × 50 cm area) facing the wall and allowed to explore freely for 5 min. We analyzed the distance walked in peripheral and center areas (40% of the total surface of the area), as well as the total number of entries into the center. As anxiety levels rise, the animal tends to remain close to walls in the peripheral zone, avoiding entry into the central zone which is considered more anxiogenic.

#### Elevated Plus Maze

The elevated plus maze was used to evaluate anxiety-related behavior in mice [51]. Mice were placed in the intersection of the four arms of the elevated plus maze and their free movement was recorded for 5 min. The elevated plus maze has two open arms and two closed arms with walls. The time spent in open and closed arms was measured, as well as the total number of arm entries made. The tendency of a subject to remain close to walls increases as anxiety levels rise, avoiding entry and the time spent in open arms.

#### Y-Maze

To evaluate non-hippocampal short-term working memory, we performed the Y-maze test as previously detailed in [29]. In brief, mice were placed in the center of a Y maze and were allowed to explore the three arms of the maze freely for 5 min. Each arm was 35 cm long, 25 cm high, and 10 cm wide and was positioned at 120° extending from a central platform. Normal animals prefer to investigate a new arm of the maze rather than the well-known one. An entry was counted when all four limbs were within the arm. A complete or incomplete arm entry was differentiated based on whether mice reached up to the end of the arm or just to the middle of the arm, respectively. A correct spontaneous alternation was considered the entry into three different arms consecutively. Finally, % spontaneous alternation was determined by dividing the number of alternations by the total number of possible alternations (the total number of arm entries minus 2) and multiplying by 100 as in [52]. Mice with less than 6 arm entries during the 5-min single trial were excluded from the analysis.

#### Object Location Memory (OLM)

We performed an OLM probe as previously detailed in [29] to evaluate spatial recognition memory depending on the hippocampus. In brief, mice were exposed to an empty area for 10 min 24 h before training. In the training phase, two identical objects (familiar) were placed in the arena, and the mouse was allowed to explore them for 5 min. To assess short-term memory, the test was conducted 90 min after training. In the test phase, one of the familiar objects was moved to a different location (novel), and then the mouse explored them again for 5 min. Time exploring both novel and familiar objects was measured and the discrimination index (DI) was calculated as follows: (time exploring the novel object – time exploring the familiar)/(time exploring novel + familiar) * 100. DI was used as a measure of the recognition of novel location and location memory, as in [53]. Animals that did not explore more than 3 s total for both objects during testing were excluded from the analysis.

### Tissue Collection and Histopathological Analyses

Animals were euthanized by cervical dislocation; brains were removed, and the right hemisphere was immediately fixed in 4% paraformaldehyde (PFA) at 4 °C overnight and embedded in paraffin for histological analyses. Five-micrometer paraffin sagittal sections were obtained by microtome and sections were deparaffinized and hydrated with deionized water. The detection of PGs by periodic acid Schiff (PAS) staining and immunohistochemical analysis were performed as detailed in [29]. For immunohistochemistry, primary and secondary antibodies used were guinea pig anti-GFAP (1:500, Synaptic Systems #173_004), rabbit anti-Iba1 (1:200, WAKO #019–19741), Alexa Fluor-conjugates [1:500, Life Technologies: goat anti-guinea pig IgG Alexa Fluor® 594 (#A11076)], and goat anti-rabbit IgG [Alexa Fluor® 488 (#A11008)]. Background controls of secondary antibodies were performed in parallel. Nuclear staining was performed with DAPI (Sigma-Aldrich). Coverslips were mounted with Fluoromount-G™ (Thermo Fisher Scientific).

### Image Acquisition and Analysis

Two sections per animal with a 24-µm-gap between them were analyzed. Three pictures per section were taken in different hippocampal areas: cornus ammonis (CA1), molecular layer of CA1 plus DG (CA1-DG), and dentate gyrus (DG). PAS-staining photomicrographs were acquired using a Leica DM RXA2 microscope for the riluzole study or using a Leica DM750 microscope (Nussloch, Germany) for resveratrol, memantine, and minocycline trials, connected to a Hamamatsu color camera with an × 40 magnification in RGB format. Immunofluorescence images were acquired using a Leica TCS Sp8 laser-scanning confocal microscope with an × 40 objective for the riluzole study or by a Leica DM6 B automatic microscope connected to a Leica k5 monochrome high sensibility camera with an × 20 objective for resveratrol, memantine, and minocycline trials. Ten to twelve *z*-axis stacks separated by 0.33 µm were taken per section and 2D reconstruction was projected as maximum intensity using ImageJ software (NIH, Bethesda, MD, USA).

For automated computer image analysis, we used the programmed tailored macros in ImageJ for PAS and fluorescence histological detection as detailed in [29].

### Data Analysis

Statistical analysis was performed with RStudio R-4.0.3 [54]. Quantitative data were represented as mean ± standard error of the mean (SEM) with a 95% confidence interval. The normality of the data was analyzed by the Shapiro–Wilk test and homogeneity of variance by the Levene test. To assess the statistical significance (*p*-value) of the effects in multiple comparisons, data with a normal distribution were analyzed by two-way ANOVA followed by a Tukey’s post hoc test. Non-parametric data were analyzed by Kruskal–Wallis followed by Dunn’s test. To assess the effect size of the interventions in multiple comparisons, Cohen’s delta coefficient (*d*) was calculated and scored as negligible (*d* < 0.20), small (*d* ≥ 0.20), medium (*d* ≥ 0.50), large (*d* ≥ 0.80), and much larger (*d* ≥ 1.00) size effect [55, 56]. A descriptive and inferential statistical summary of analyzed behavioral and histopathological variables is supplied (see Supplementary Table S1 and Table S2). A critical value for significance of *p < 0.05 was used throughout the study.

## Results

In this work, we have evaluated the efficacy of the treatment of *Epm2b − / − *mice with four drugs, two of them previously used as glutamatergic modulators (riluzole and memantine) and the other two used as neuroinflammatory-modifying therapeutic agents (resveratrol and minocycline). Treatments were administered in male mice of 3 months of age (corresponding to an early stage of LD) for 2 months. After treatments, we performed an in vivo analysis of anxiety-like, cognitive behavior and neurodegenerative signs followed by an ex vivo histopathological analysis of PG inclusions, astrogliosis, and microgliosis in the corresponding mouse brains. For the sake of clarity, we present the results as independent treatments (although memantine and minocycline were performed at the same time since they shared the same vehicle), comparing the values of treated *Epm2b − / − *mice to *Epm2b − / − *mice that received only water, vehicle, or saline, respectively.

### Decreased Anxiety-like and Hyperactive Behavior of Epm2b − / − Mice Are Attenuated by Anti-neuroinflammatory Treatments

**Fig. 1:**
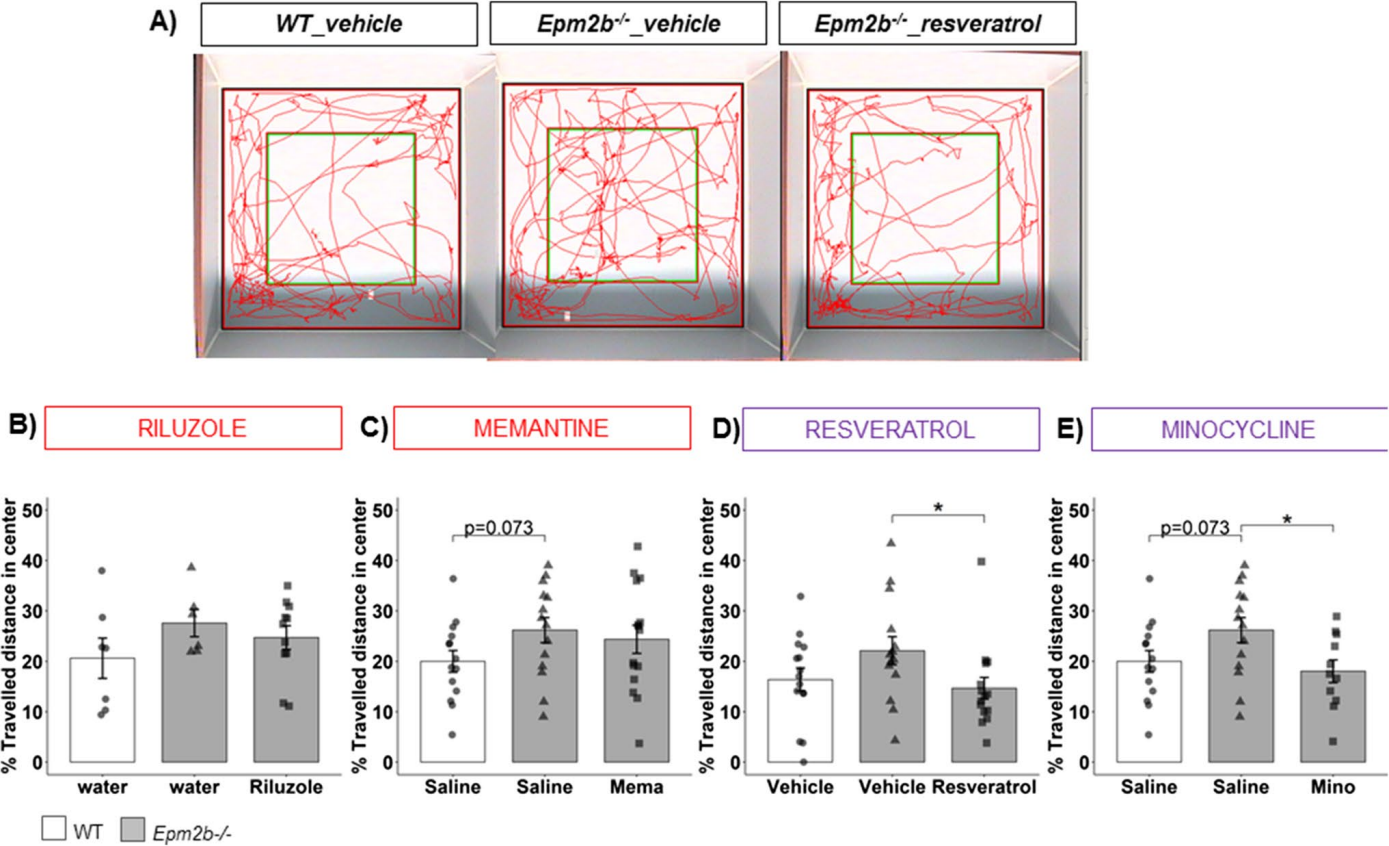
Anxiety-like behavior state in *Epm2b − / − *mice and the therapeutic efficacy of riluzole, memantine, resveratrol, and minocycline treatments. **A** Representative tracks were recorded with SMART video system to evaluate the anxiety-like behavior as measured by Open field (see “Materials and Methods” section). **B**–**E** % traveled distance in the center representing the anxiety levels of each animal. Bar graphs show mean ± standard error of the mean (SEM). Individual data points and the comparisons between groups which were *p* < 0.1 are indicated. Depending on the normal distribution and homoscedasticity of the data, statistical differences were analyzed, by two-way ANOVA following a Tukey’s post hoc to multiple comparisons in (**B**) and by one-way Kruskal–Wallis following a Dunn’s post hoc test to multiple comparisons in (**C**–**E**). Statistical significance was defined as *p < 0.05. A summary of all descriptive (mean ± SEM) and inferential data (all comparisons between groups) is available in Table S1. Treatments related to glutamatergic transmission are boxed in red and those related to neuroinflammation are in magenta

The anxiety-like behavior was evaluated in *Epm2b − / − *mice at 5 months of age by carrying out open field and elevated plus maze tests. In the open file test, the percentage of traveled distance in the center and the number of entries into the center were measured as key indicators of anxiety and hyperactivity, respectively. Untreated *Epm2b − / − *mice showed no statistical differences respect to WT in traveling in the center zone (Fig. 1) [e.g., *Epm2b − / − *(26.17 ± 2.49) and WT (19.97 ± 2.13, *p* = 0.073, *d* =  − 0.71 medium) (Fig. 1C)] or making a different number of entries into the center [e.g., *Epm2b − / − *(21.92 ± 2.53) and WT (16.92 ± 2.19, *p* = 0.239, *d* =  − 0.56 medium) (Table S1)]. After the anti-inflammatory treatments (resveratrol and minocycline), the % traveled distance of *Epm2b − / − *in the center was decreased significantly by resveratrol (14.64 ± 2.15, *p* = 0.011*, *d* = 0.80 large) (Fig. 1D) and minocycline (18.01 ± 2.24, *p* = 0.032*, *d* = 0.95 large) (Fig. 1E), in comparison to the corresponding *Epm2b − / − *mice treated with the respective vehicle, suggesting an amelioration in the activity of the treated animals (Fig. 1D and 1E) (Table 1). In contrast, riluzole (24.71 ± 2.34, *p* = 0.896, *d* = 0.38 small) (Fig. 1B) and memantine (24.36 ± 2.79, *p* = 0.593, *d* = 0.17 negligible) (Fig. 1C) had no effect on this parameter (Table S1). Regarding the number of entries into the center, only minocycline was capable to decrease this parameter significantly (9.63 ± 1.47, *p* = 0.0017**, *d* = 1.57 much large) in comparison to untreated *Epm2b − / − *animals (Table S1).

**Table 1:** Summary of selected comparisons of *Epm2b − / − *mice treated or not with the indicated compounds (see Supplementary Tables S1 and S2)

Behavioral or histological features	*Epm2b − / − *^*#*^	*Epm2b − / − * _riluzol*e*	*Epm2b − / − *_memantine	*Epm2b − / − *_resveratrol	*Epm2b − / − *_minocycline
**Anxiety** Openfield: % traveled distance in center	Not modified *p* = 0.073/*d* medium	Not modified *p* = 0.896/*d* small	Not modified *p* = 0.593/*d* negligible	**Ameliorated** *p* = 0.011*/*d* large	**Ameliorated** *p* = 0.032*/*d* large
**Incomplete exploration behavior** y-maze: % incomplete alternancies	**Increased** *p* = 0.017*/*d* large	Not modified *p* = 0.834/*d* small	**Ameliorated** *p* = 0.035*/*d* medium	Not modified *p* = 0.992/*d* negligible	Not modified *p* = 0.078/*d* medium
**Hiperactivity** OLM: activity time (s)	**Increased** *p* = 0.048*/*d* medium	Not modified *p* = 0.506/*d* large	**Ameliorated** *p* = 0.016*/*d* large	Not modified *p* = 0.920/*d* negligible	Not modified *p* = 0.331/*d* medium
**Neurodegenerative signs** Hindlimb clasping score	**Increased** *p* = 9.97e − 08****	**Worsening** *p* = 2.14e − 10****	**Ameliorated** *p* = 0.0007***	**Ameliorated** *p* = 1.63e − 07****	**Ameliorated** *p* = 4.73e − 09****
**PG inclusions** PAS number	**Increased** *p* = 0.006**/*d* much large	Not modified *p* = 0.260/*d* much large	Not modified *p* = 0.264/*d* large	Not modified *p* = 0.864/*d* negligible	Not modified *p* = 0.780/*d* negligible
**Astrogliosis** % GFAP area	**Increased** *p* = 0.006**/*d* much large	Not modified *p* = 0.643/*d* negligible	Not modified *p* = 0.369/*d* small	Not modified *p* = 0.921/*d* small	Not modified *p* = 0.903/*d* small
**Microgliosis** % Iba1 + cells	Not modified *p* = 0.543/*d* large	Not modified *p* = 0.090/*d* much large	Not modified *p* = 0.988/*d* negligible	Not modified *p* = 0.962/*d* small	Not modified *p* = 0.975/*d* small

^#^Comparison of *Epm2b − / − *mice with control animals treated with saline (from the memantine and minocycline trials). Statistical significance is considered *p < 0.05, **p < 0.01, ***p < 0.001, ****p < 0.0001. Comparisons with a *p*-value < 0.05 are shown in bold. Cohen’s delta coefficient (*d*) is indicated as negligible (*d* < 0.20), small (*d* > 0.20), medium (*d* > 0.50), large (*d* > 0.80), and much large (*d* > 1.00) size effect (see “Materials and Methods” section)

We used the elevated plus maze as an alternative method to assess the effectiveness of the different treatments on the anxiety-like behavior in *Emp2b − / − *mice. None of the treatments were able to modify the *Epm2b − / − *mice behavior in the elevated plus maze test (Table S1), except for minocycline which significantly decreased the number of entries into arms (25.33 ± 3.33, *p* = 0.050*, *d* = 0.87 large) compared to *Epm2b − / − *treated with the corresponding vehicle*.* Therefore, minocycline was the only effective treatment to ameliorate the behavior of *Epm2b − / − *mice in both anxiety tests, open field and elevated plus maze.

### Attention Defect in Epm2b − / − Mice Is Improved by Memantine and Minocycline Treatments

**Fig. 2:**
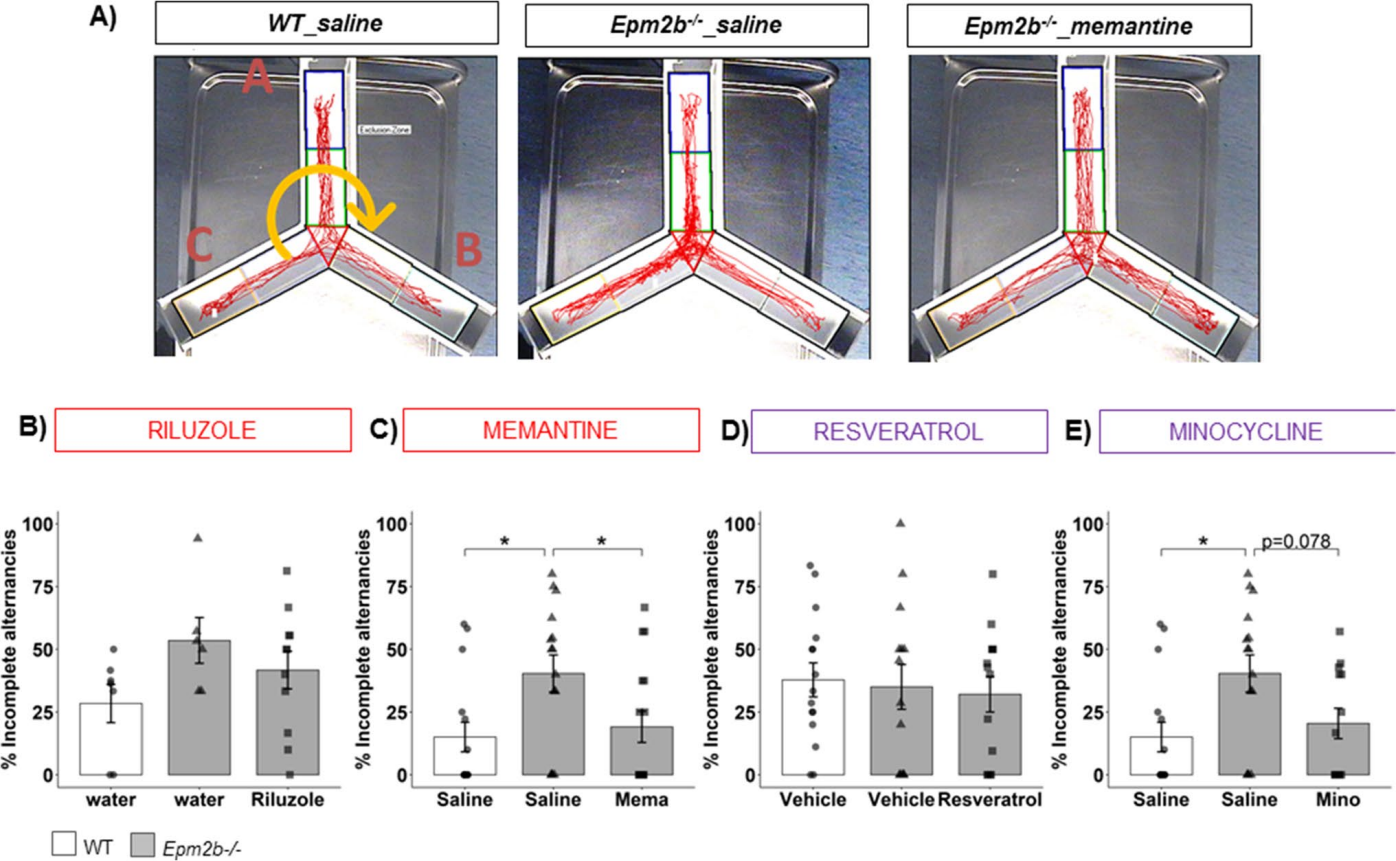
Cognitive state in *Epm2b − / − *mice and the therapeutic efficacy of riluzole, memantine, resveratrol, and minocycline treatments. **A** Representative tracks were recorded with SMART video system to evaluate the working memory as measured by y-maze (see “Materials and Methods” section). **B**–**E** % of the incomplete alternations representing the attention of staying on the task of each animal. Bar graphs show mean ± standard error of the mean (SEM). Individual data points and the comparisons between groups which were *p* < 0.1 are indicated. Depending on the normal distribution and homoscedasticity of the data, statistical differences were analyzed, by two-way ANOVA following a Tukey’s post hoc to multiple comparisons in (**B**, **D**) and by one-way Kruskal–Wallis following a Dunn’s post hoc test to multiple comparisons in (**C**, **E**). Statistical significance was defined as *p < 0.05. A summary of all descriptive (mean ± SEM) and inferential data (all comparisons between groups) is available in Table S1

The cognitive profile of *Epm2b − / − *mice was evaluated at 5 months of age by assessing working memory and short-term location memory. To evaluate working memory, animals were tested for the % of spontaneous alternations in the Y-maze (Table S1), and % of incomplete alternations were quantified (Fig. 2). Regarding the % of spontaneous alternations, although a repeated tendency to a slight decrease of this parameter in untreated *Epm2b − / − *mice compared to WT was present through the trials (Table S1), we concluded that *Epm2b − / − *mice did not display any working memory defect due to the absence of either statistical significance (*p*-value) or large effect size (*d*) in the means, in agreement with previously published results [29]. In contrast, the % of incomplete alternations was increased in untreated *Epm2b − / − *compared to WT mice (Fig. 2) (e.g., 40.38 ± 7.40 in *Epm2b − / − *mice vs. 15.03 ± 5.90, in WT mice; *p* = 0.017*, *d* =  − 0.97 large; Fig. 2C), suggesting an attention defect in ending the task. Interestingly, the % of incomplete alternations in *Epm2b − / − *mice was significantly decreased by memantine (19.11 ± 6.18, *p* = 0.035*, *d* = 0.80 medium), suggesting a positive effect of this drug in improving the staying on-task of exploration (Table 1). Treatments with riluzole, resveratrol, or minocycline did not ameliorate significantly the results of the untreated *Epm2b − / − *mice (Fig. 2B, 2D, and 2E) (Table S1).

**Fig. 3:**
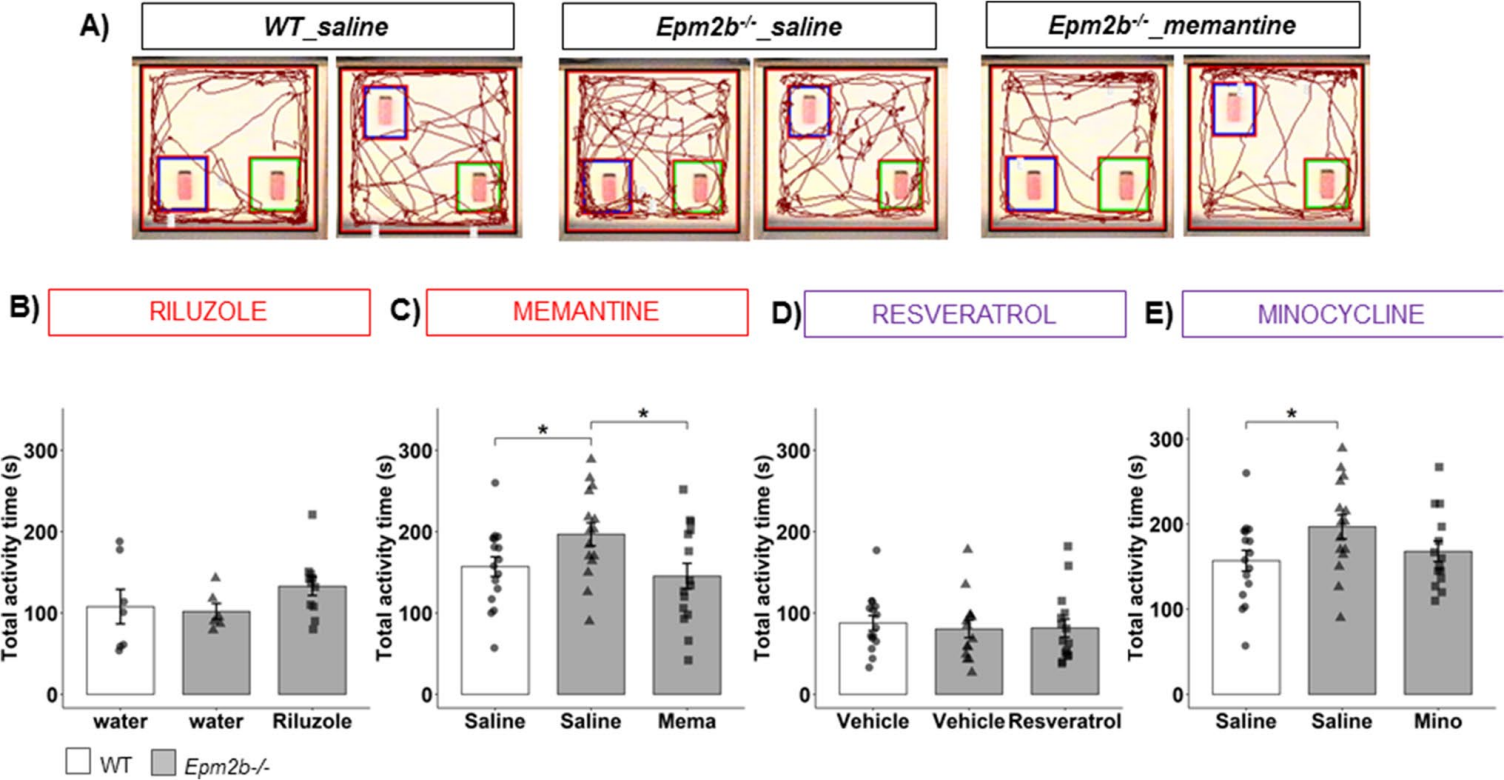
Hyperactivity in *Epm2b − / − *mice and the therapeutic efficacy of riluzole, memantine, resveratrol, and minocycline treatments. **A** Representative tracks were recorded with SMART video system to evaluate the spatial location memory as measured by OLM (see “Materials and Methods” section). **B**–**E** Total activity time representing the activity levels of each animal. Bar graphs show mean ± standard error of the mean (SEM). Individual data points and the comparisons between groups which were *p* < 0.1 are indicated. Depending on the normal distribution and homoscedasticity of the data, statistical differences were analyzed, by two-way ANOVA following a Tukey’s post hoc to multiple comparisons in (**B**) and by one-way Kruskal–Wallis following a Dunn’s post hoc test to multiple comparisons in (**C**–**E**). Statistical significance was defined as *p < 0.05. A summary of all descriptive (mean ± SEM) and inferential data (all comparisons between groups) is available in Table S1

In relation to hippocampal memory, we studied spatial short-term memory using the object location memory test (OLM) (Fig. 3). The discrimination index (DI) of object location and the total activity time were measured. There were no significant differences (*p*-value) or remarkable effect size (*d*) in DI among all the groups (Table S1), suggesting that short-term location memory was not affected in *Epm2b − / − *mice at 5 months of age, in agreement with previous results [29]. However, we noticed an increase in the total activity time in *Emp2b − / − *compared to WT in untreated animals (e.g., 196.86 ± 14.10 s in *Epm2b − / − *mice vs. 157.00 ± 12.22 s in WT mice; *p* = 0.048*, *d* =  − 0.77 medium; Fig. 3C), highlighting again the hyperactive behavior in *Epm2b − / − *mice. Among the treatments, we nicely observed that only memantine treatment significantly decreased the total activity time in *Epm2b − / − *mice (145.26 ± 15.73 s, *p* = 0.016*, *d* =  − 0.89 large) (Fig. 3C), reducing hyperactive behavior (Table 1). Again, treatments with riluzole, resveratrol, or minocycline did not ameliorate the results of the untreated *Epm2b − / − *mice (Fig. 3B, 3D, and 3E) (Table S1).

### Neurodegenerative Signs Detected in 5-month-old Epm2b − / − Mice Are Ameliorated by Memantine, Resveratrol, and Minocycline Treatments

**Fig. 4:**
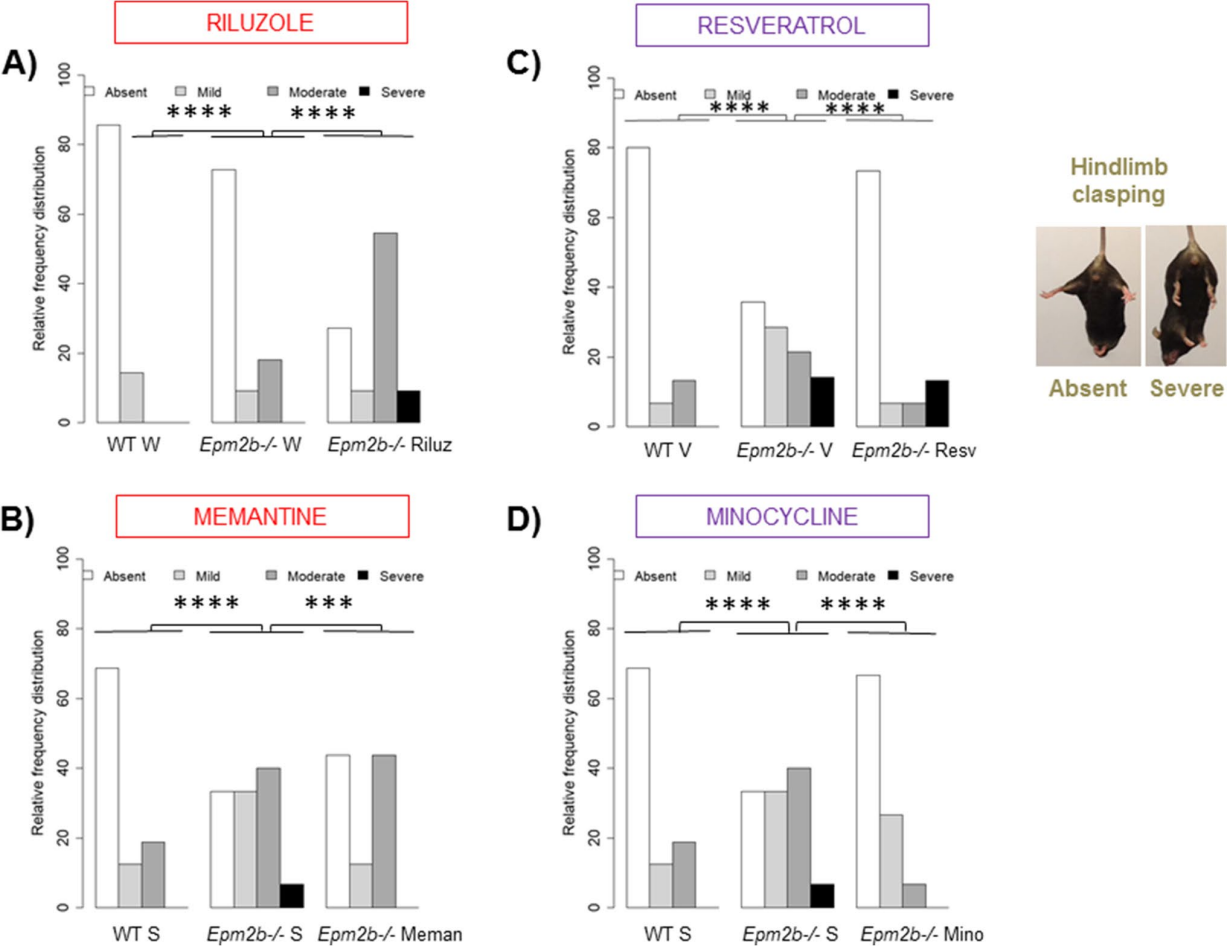
Neurodegenerative state in *Epm2b − / − *mice and the therapeutic efficacy of riluzole, memantine, resveratrol, and minocycline treatments. **A**–**D** Relative frequency distribution of the hindlimb clasping score representing the severity of neurodegenerative signs. Frequency histograms show frequency distribution among 4 scores: absent, mild, moderate, and severe. Statistical differences between groups were analyzed by Pearson’s chi-square test or by Fisher’s exact test when sample sizes were zero. Statistical significance was defined as ***p < 0.001 and ****p < 0.0001. A summary of all contingency tables and inferential data (all comparisons between groups) is available in Table S2

*Epm2b − / − *mice were evaluated for abnormal postures related to neurodegeneration by using the hindlimb clasping test. In untreated *Epm2b − / − *mice, at 5 months of age, the hindlimb clasping score was significantly and repeatedly worse in all trials compared to WT (Fig. 4A–D) (Table S2). Among the treatments, we observed a significant improvement after memantine (*p* = 0.0007***) (Fig. 4B), resveratrol (*p* = 1.63e − 07****) (Fig. 4C), and minocycline (*p* = 4.73e − 09****) (Fig. 4D) treatments. Therefore, neurodegenerative signs present in *Epm2b − / − *mice were ameliorated by these pharmacological treatments (Table 1). However, riluzole treatment worsened the neurodegenerative signs (*p* = 2.14e − 10****) (Fig. 4A) (Table S2).

### Riluzole, Memantine, Resveratrol, and Minocycline Do Not Have Any Effect on the Formation of Polyglucosan Inclusions in Epm2b − / − Mice

**Fig. 5:**
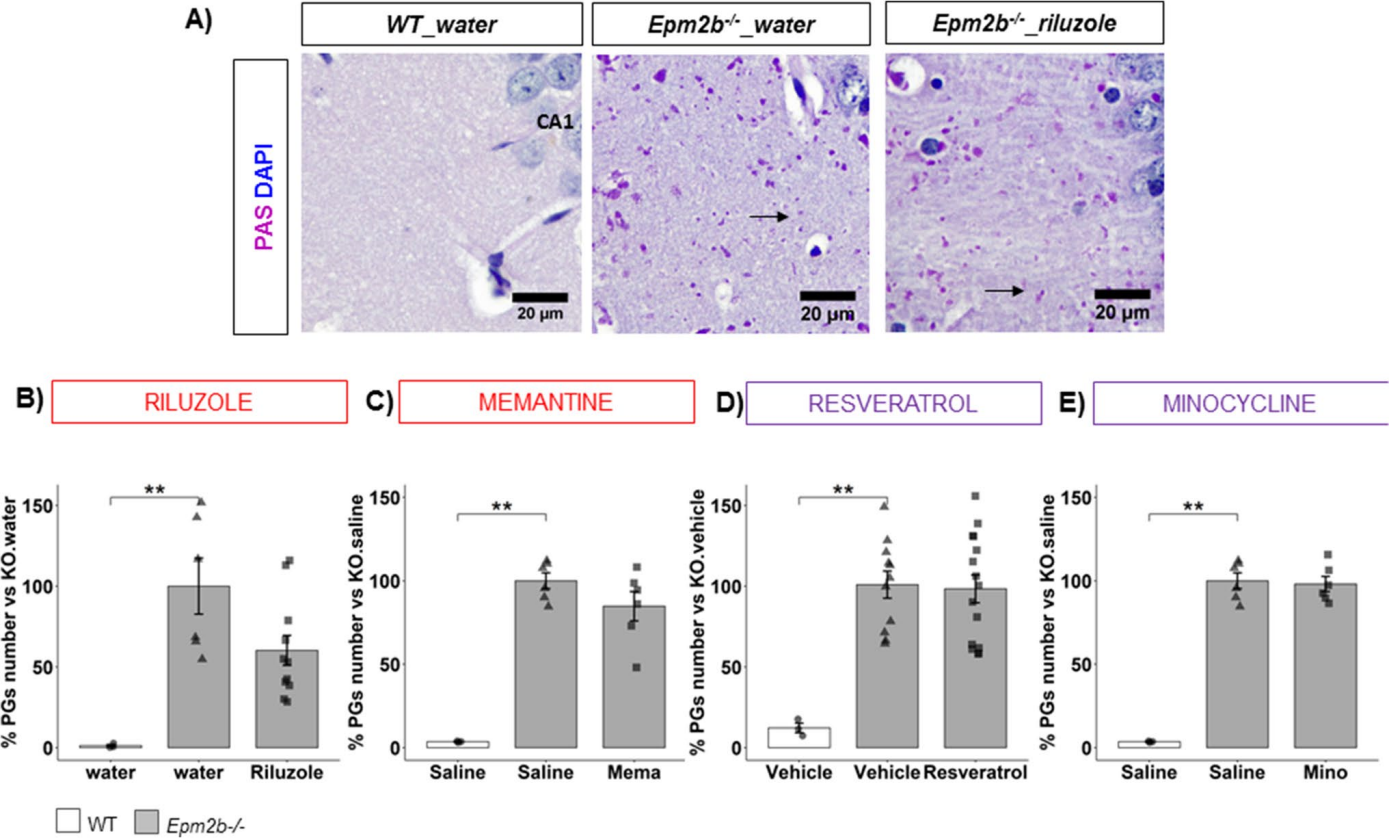
Accumulation of PGs in the hippocampus of *Epm2b − / − *mice and the therapeutic efficacy of riluzole, memantine, resveratrol, and minocycline. **A** Representative microscopy images of PG detection (in pink; see also black arrows) in CA1 region of the hippocampus by PAS staining; neural nuclei are in blue. **B**–**E** % of PG number versus the number present in untreated *Epm2b − / − *mice. Bar graphs show mean ± standard error of the mean (SEM). Individual data points and the comparisons between groups which were *p* < 0.1 are indicated. Depending on the normal distribution and homoscedasticity of the data, statistical differences were analyzed by one-way ANOVA with Welch’s correction following a Tukey’s post hoc to multiple comparisons in (**B**) and by one-way Kruskal–Wallis following a Dunn’s post hoc test to multiple comparisons in (**C**–**E**). Statistical significance was defined as **p < 0.01. A summary of all descriptive (mean ± SEM) and inferential data (all comparisons between groups) is available in Table S1. Scale 20 µm

To evaluate the presence of PG inclusions in mice, brain slices were obtained and stained with a periodic acid-Schiff stain (PAS) which detects polysaccharides such as glycogen. The number of PGs per 10,000 µm2 was quantified by image analysis as indicated in the “Materials and Methods” section and the percentages of PG number vs. untreated *Epm2b − / − *mice were plotted on a graph. Representative pictures of PAS staining (Fig. 5A) disclosed an enormous number of PGs in *Epm2b − / − *compared to WT mice (e.g., 100.00 ± 17.31 in *Epm2b − / − *mice vs. 1.18 ± 0.77 in WT mice; *p* = 0.0099**, d =  − 2.75 much large; Fig. 5B), which was significantly repeated through all trials (Fig. 5C–E) (Table S1). This greater number of PGs was not significantly ameliorated by riluzole (60.23 ± 9.26, *p* = 0.260, *d* = 1.13 much large) or by memantine (84.76 ± 8.79, *p* = 0.264, *d* = 0.88 large) treated *Epm2b − / − *mice (Fig. 5B-C) compared to *Epm2b − / − *mice treated with the corresponding vehicle (Table 1). Thus, we consider that 2-month glutamatergic treatments might have, if any, only a minor effect in preventing the formation of PG inclusions in *Epm2b − / − *mice. In the same way, anti-neuroinflammatory treatments did not affect PG accumulation either (resveratrol 98.34 ± 8.58, *p* = 0.864, *d* = 0.05 negligible; minocycline 98.03 ± 4.51, *p* = 0.780, *d* = 0.17 negligible) (Fig. 5D,E) (Table S1).

### Reactive Astrogliosis in Epm2b − / − Mice Is Not Modulated by Any of the Pharmacological Treatments

**Fig. 6:**
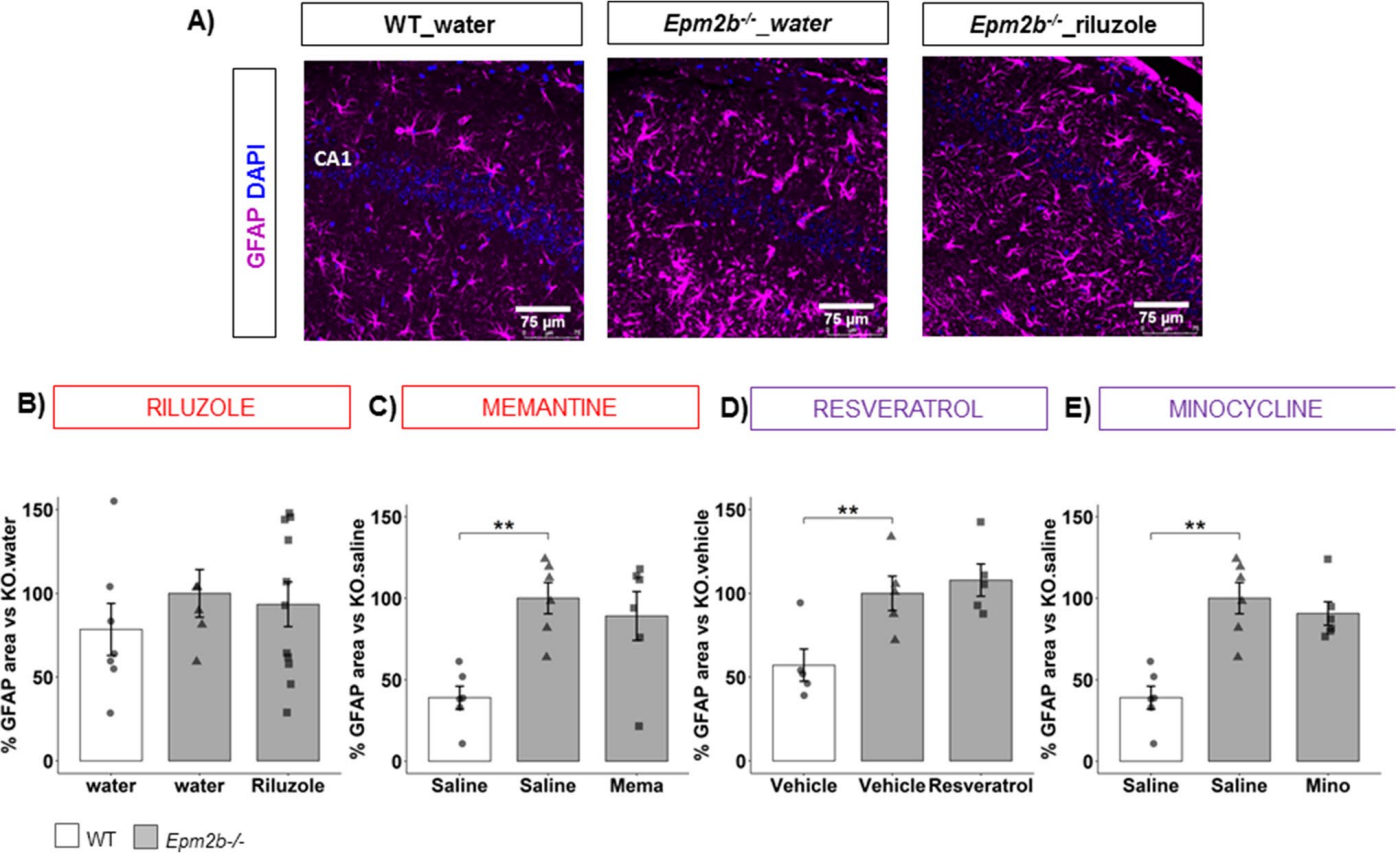
Effect of the different treatments on the reactive astrogliosis of the *Epm2b − / − *mice. **A** Representative immunofluorescence confocal micrographs of the CA1 region of the hippocampus. Astrocytes (GFAP staining) are in magenta and DAPI staining of cellular nuclei are in blue. **B**–**E** % GFAP area related to the value obtained in untreated *Epm2b − / − *mice, representing the extension of the reactive astrogliosis in the hippocampus. Bar graphs show mean ± standard error of the mean (SEM). Individual data points and the comparisons between groups which were *p* < 0.1 are indicated. Depending on the normal distribution and homoscedasticity of the data, statistical differences were analyzed by two-way ANOVA following a Tukey’s post hoc to multiple comparisons in (**D**) and by one-way Kruskal–Wallis following a Dunn’s post hoc test to multiple comparisons in (**B**, **C**, **E**). Statistical significance was defined as **p < 0.01. A summary of all descriptive (mean ± SEM) and inferential data (all comparisons between groups) is available in Table S1. Scale 75 µm

Since we have found that the accumulation of PGs is significantly correlated to the appearance of reactive astroglia and microglia in *Epm2b − / − *mice [29], we examined reactive astrogliosis in *Epm2b − / − *mice. We detected the astrocytic marker GFAP (in magenta) and the nuclear marker DAPI (in blue) by immunofluorescence (Fig. 6A). As described previously [21, 28, 29], untreated *Epm2b − / − *showed a massive GFAP + area compared to WT mice (Fig. 6A) (e.g., 100.00 ± 9.57 in *Epm2b − / − *mice vs. 38.98.15 ± 7.05 in WT mice; *p* = 0.0062**, *d* =  − 2.96 much large; Fig. 6C) (Table 1), which suggests a remarkable pathological reactive astrogliosis in the brain of *Epm2b − / − *mice. Unfortunately, neither riluzole (Fig. 6B), memantine (Fig. 6C), resveratrol (Fig. 6D), nor minocycline (Fig. 6E) treatment significantly modified the astrogliosis in the brain of *Epm2b − / − *mice (Table S1).

### Riluzole, Memantine, Resveratrol, and Minocycline Do Not Have Any Effect on the Microgliosis Present in Epm2b − / − Mice

**Fig. 7:**
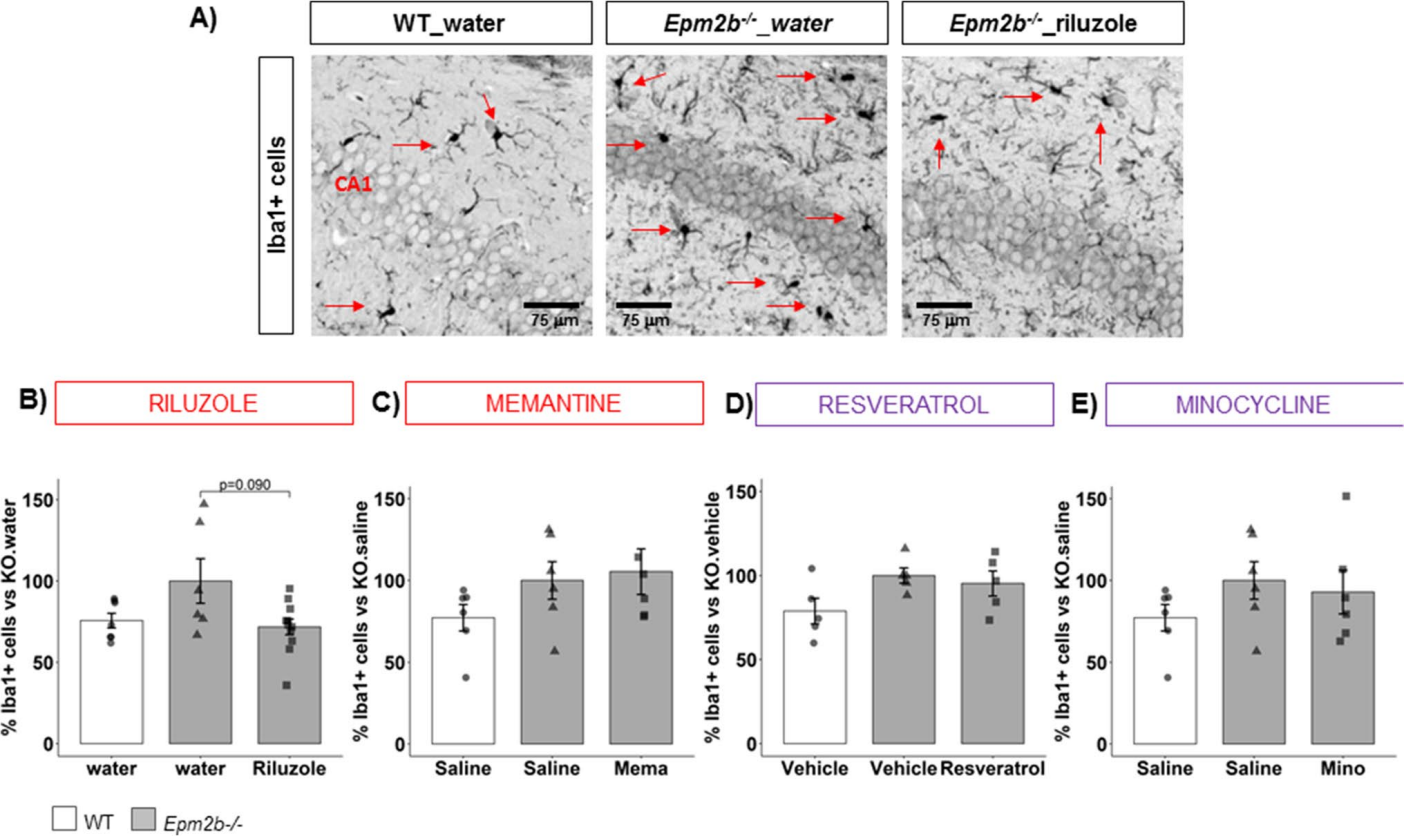
Effect of the different treatments on the reactive microglia in the hippocampus of *Epm2b − / − *mice. **A** Representative immunofluorescence confocal micrographs of Iba1 (in gray) to appreciate the remarkable morphological changes in microglia in *Epm2b − / − *mice (see red arrows); **B**–**E %** Iba1 positive cells related to the value obtained in untreated *Epm2b − / − *mice*,* representing the extension of the reactive microglia in the hippocampus with morphological changes. Bar graphs show mean ± standard error of the mean (SEM). Individual data points and the comparisons between groups which were *p* < 0.1 are indicated. Depending on the normal distribution and homoscedasticity of the data, statistical differences were analyzed by two-way ANOVA following a Tukey’s post hoc to multiple comparisons in (**C**–**E**) and by one-way Kruskal–Wallis following a Dunn’s post hoc test to multiple comparisons in (**B**). A summary of all descriptive (mean ± SEM) and inferential data (all comparisons between groups) is available in Table S1. Scale 75 µm

Finally, we detected the microglial marker Iba1 (in gray) by immunofluorescence (Fig. 7A) and the number of Iba1 + cells with clear changes in morphology was counted as a marker of microglial activation [57]. However, in our settings, we were not able to detect statistically significant differences between untreated *Epm2b − / − *(100.00 ± 11.48) and WT (77.18 ± 4.49, *p* = 0.543, *d* =  − 0.94 large) (Fig. 7) (Table S1). None of the treatments showed any significant effect in comparison to untreated *Epm2b − / − *animals (Fig. 7A–E) (Table S1).

## Discussion

Lafora disease (LD) is a fatal rare neurological disorder that leads to the death of patients around 10 years from onset. Work is underway to develop drugs that could be used as a cure or as disease-modifying agents. These strategies have been aimed to reduce the formation of LBs by inhibiting glycogen synthase (GYS1) activity, either by using antisense oligonucleotides (ASOs) [58] or by small chemical compounds that inhibit GYS1 [59]. Moreover, one approach was designed to digest LBs by using an antibody-enzyme fusion [60, 61]. In addition, alternative strategies aimed to ameliorate the symptoms of LD have been implemented in LD mouse models. For example, a ketogenic diet has been recently described as being useful in reducing the formation of LBs [62], and our group has described the use of repurposing drugs that ameliorate LD pathophysiology. In this sense, we reported that metformin has a beneficial effect on *Epm2b − / − *mice [28] and these results allowed the designation of this compound as an orphan drug for the treatment of LD, both by the European Medicines Agency (EMA/3/16/1803) and the Food and Drug Administration (FDA/#17–6161). Recently, we described that the use of modulators of neuroinflammation had also a beneficial effect in *Epm2b − / − *mice, especially propranolol, which reduced the formation of reactive glia and had amelioration of different behavioral tests [29].

In this work, we have expanded our analysis to additional repurposing drugs focusing our attention on compounds that affect either glutamatergic transmission or neuroinflammation. As an example of the first, we used riluzole and memantine. Riluzole is the first FDA-approved medication for amyotrophic lateral sclerosis (ALS) due to its capability to modulate glutamate neurotransmission, not only by inhibiting presynaptic glutamate release but also by enhancing the clearance of this neurotransmitter by astrocytic glutamate transporters, which results in a reduction of postsynaptic glutamate receptor signaling [30]. We found that riluzole did not have a major effect on the behavioral tests described in this study, except that it worsened the neurodegenerative-related hindlimb clasping test. At the histopathological level, riluzole did not modify significantly any of the tested histopathological parameters (Table 1).

In the case of memantine, an antagonist of postsynaptic glutamate NMDA receptors, which reduces the effects of excitotoxic glutamate release, we found a statistically significant beneficial effect of this drug on several behavioral tests related to exploration behavior (incomplete alternancies in Y-maze), hyperactivity (OLM activity time), and neurodegenerative signs (hindlimb clasping). These beneficial effects were not related to the levels of PGs or reactive glia (Table 1), suggesting that the positive effect of memantine could be due to the inhibition of post-synaptic NMDA receptor signaling. It is worth pointing out that the excitotoxicity associated with the overactivation of this receptor may cause neuronal death and cognitive deficits associated with dementia such as learning and memory impairments [35]. Moreover, in several clinical assays, memantine monotherapy was found to exert efficacy on hyperactivity and attention deficit in adult patients with attention deficit hyperactivity disorder (ADHD) [63]. Therefore, the beneficial effects of memantine on the cognitive and behavioral profile of *Epm2b − / − *mice are likely due to an amelioration of the excitotoxicity produced by imbalanced glutamate levels present in these mice [24]. In this sense, the effects of memantine would be analogous to the beneficial effects obtained with perampanel, an inhibitor of post-synaptic glutamate AMPA receptors [64]. Probably, the beneficial effects of both compounds are attributed to a decrease in neuronal hyperexcitability due to a downregulation of the activity of the postsynaptic glutamate receptors AMPA (by perampanel) [65] and NMDA (by memantine) (this work).

To modulate neuroinflammation, we used resveratrol and minocycline. It has been reported that resveratrol has anti-oxidative, anti-aging, and anti-inflammatory properties [36, 37]. In *Epm2b − / − *mice, this compound had a beneficial effect on some behavioral tests related to anxiety (open field) and neurodegenerative signs (Hindlimb clasping). However, resveratrol did not affect the levels of PGs or the levels of reactive glia (Table 1). Perhaps, the beneficial effect of resveratrol could be related to alternative pathways, since it has been described that resveratrol decreases the production of pro-inflammatory cytokines via the activation of AMP-activated protein kinase (AMPK), SIRT1, and SOCS1, and also reduces reactive oxygen species (ROS) production [66, 67].

Finally, we used minocycline, a tetracycline antibiotic derivative that has anti-inflammatory and neuroprotective activities in several neurodegenerative conditions [40, 41]. In *Epm2b − / − *mice we found a beneficial effect of this compound on different behavioral tests related to anxiety (open field) and neurodegenerative signs (hindlimb clasping). However, this drug did not affect the levels of PGs and, surprisingly, it did not reduce the levels of reactive glia (Table 1), suggesting that minocycline had affected alternative pathways. In this sense, it is worth noting that, in addition to its anti-inflammatory properties, minocycline prevents neurons from glutamate toxicity as this compound reduces the release of glutamate and the excitability of neurons in the hippocampus [68], represses the expression of the NR2A subunit of the NMDA receptor [47], attenuates NMDA-induced Ca +  + entry and excitotoxicity [69], and ameliorated downregulation of glial glutamate transporter expression promoting glutamate uptake in the spinal sensory synapses [70]. Therefore, the beneficial effects of minocycline on the cognitive and behavioral profile of *Epm2b − / − *mice are likely due to an amelioration of neuronal excitotoxicity, resembling the memantine treatment described above.

In summary, among the four different compounds analyzed in this work, selected to modify either the altered glutamatergic transmission or the neuroinflammation profiles present in *Epm2b − / − *mice, memantine (an inhibitor of the post-synaptic NMDA receptors) and minocycline (an antibiotic derivative with broad physiological functions) were the most promising candidates to be considered, either alone or in combination with other repurposing drugs, in future therapeutic strategies for LD. It is interesting to point out that, despite targeting initially both pathways separately, we obtained interlinked pharmacological effects between them, mainly related to amelioration of glutamate-induced excitotoxicity. This highlights the importance of the altered glutamatergic transmission in the development of the pathophysiological symptoms of LD.

## Supplementary Information

Below is the link to the electronic supplementary material.

Supplementary file1 (PDF 219 KB)

## Data Availability

All data generated or analyzed during this study are included in this published article and its supplementary information files.

## References

[1] Turnbull J, Tiberia E, Striano P, Genton P, Carpenter S, Ackerley CA, Minassian BA (2016). Lafora disease. Epileptic Disord.

[2] Lafora GR, Glueck B (1911). Beitrag zur histogpathologie der myoklonischen epilepsie. Gesamte Neurol Psychiatr.

[3] Sakai M, Austin J, Witmer F, Trueb L (1970). Studies in myoclonus epilepsy (Lafora body form). II. Polyglucosans in the systemic deposits of myoclonus epilepsy and in corpora amylacea. Neurology.

[4] Mitra S, Gumusgoz E, Minassian BA (2022). Lafora disease: current biology and therapeutic approaches. Rev Neurol (Paris).

[5] Markussen KH, Macedo JKA, Machio M, Dolce A, Goldberg YP, Vander Kooi CW, Gentry MS (2021). The 6th International Lafora Epilepsy Workshop: advances in the search for a cure. Epilepsy Behav.

[6] Minassian BA, Lee JR, Herbrick JA, Huizenga J, Soder S, Mungall AJ, Dunham I, Gardner R (1998). Mutations in a gene encoding a novel protein tyrosine phosphatase cause progressive myoclonus epilepsy. Nat Genet.

[7] Serratosa JM, Gomez-Garre P, Gallardo ME, Anta B, de Bernabe DB, Lindhout D, Augustijn PB, Tassinari CA (1999). A novel protein tyrosine phosphatase gene is mutated in progressive myoclonus epilepsy of the Lafora type (EPM2). Hum Mol Genet.

[8] Chan EM, Young EJ, Ianzano L, Munteanu I, Zhao X, Christopoulos CC, Avanzini G, Elia M (2003). Mutations in NHLRC1 cause progressive myoclonus epilepsy. Nat Genet.

[9] Garcia-Gimeno MA, Knecht E, Sanz P (2018). Lafora disease: a ubiquitination-related pathology. Cells.

[10] Nitschke F, Ahonen SJ, Nitschke S, Mitra S, Minassian BA (2018). Lafora disease - from pathogenesis to treatment strategies. Nat Rev Neurol.

[11] Ganesh S, Delgado-Escueta AV, Sakamoto T, Avila MR, Machado-Salas J, Hoshii Y, Akagi T, Gomi H (2002). Targeted disruption of the Epm2a gene causes formation of Lafora inclusion bodies, neurodegeneration, ataxia, myoclonus epilepsy and impaired behavioral response in mice. Hum Mol Genet.

[12] DePaoli-Roach AA, Tagliabracci VS, Segvich DM, Meyer CM, Irimia JM, Roach PJ (2010). Genetic depletion of the malin E3 ubiquitin ligase in mice leads to lafora bodies and the accumulation of insoluble laforin. J Biol Chem.

[13] Turnbull J, Wang P, Girard JM, Ruggieri A, Wang TJ, Draginov AG, Kameka AP, Pencea N (2010). Glycogen hyperphosphorylation underlies lafora body formation. Ann Neurol.

[14] Criado O, Aguado C, Gayarre J, Duran-Trio L, Garcia-Cabrero AM, Vernia S, San Millan B, Heredia M (2012). Lafora bodies and neurological defects in malin-deficient mice correlate with impaired autophagy. Hum Mol Genet.

[15] Garcia-Cabrero AM, Marinas A, Guerrero R, de Cordoba SR, Serratosa JM, Sanchez MP (2012). Laforin and malin deletions in mice produce similar neurologic impairments. J Neuropathol Exp Neurol.

[16] Valles-Ortega J, Duran J, Garcia-Rocha M, Bosch C, Saez I, Pujadas L, Serafin A, Canas X (2011). Neurodegeneration and functional impairments associated with glycogen synthase accumulation in a mouse model of Lafora disease. EMBO Mol Med.

[17] Taneja K, Ganesh S (2021). Dendritic spine abnormalities correlate with behavioral and cognitive deficits in mouse models of Lafora disease. J Comp Neurol.

[18] Ortolano S, Vieitez I, Agis-Balboa RC, Spuch C (2014). Loss of GABAergic cortical neurons underlies the neuropathology of Lafora disease. Mol Brain.

[19] Rubio-Villena C, Viana R, Bonet J, Garcia-Gimeno MA, Casado M, Heredia M, Sanz P (2018). Astrocytes: new players in progressive myoclonus epilepsy of Lafora type. Hum Mol Genet.

[20] Auge E, Pelegri C, Manich G, Cabezon I, Guinovart JJ, Duran J, Vilaplana J (2018). Astrocytes and neurons produce distinct types of polyglucosan bodies in Lafora disease. Glia.

[21] Lopez-Gonzalez I, Viana R, Sanz P, Ferrer I (2017). Inflammation in Lafora disease: evolution with disease progression in laforin and Malin knock-out mouse models. Mol Neurobiol.

[22] Lahuerta M, Gonzalez D, Aguado C, Fathinajafabadi A, Garcia-Gimenez JL, Moreno-Estelles M, Roma-Mateo C, Knecht E (2020). Reactive glia-derived neuroinflammation: a novel hallmark in Lafora progressive myoclonus epilepsy that progresses with age. Mol Neurobiol.

[23] Munoz-Ballester C, Berthier A, Viana R, Sanz P (2016). Homeostasis of the astrocytic glutamate transporter GLT-1 is altered in mouse models of Lafora disease. Biochim Biophys Acta.

[24] Munoz-Ballester C, Santana N, Perez-Jimenez E, Viana R, Artigas F, Sanz P (2019). In vivo glutamate clearance defects in a mouse model of Lafora disease. Exp Neurol.

[25] Perez-Jimenez E, Viana R, Munoz-Ballester C, Vendrell-Tornero C, Moll-Diaz R, Garcia-Gimeno MA, Sanz P (2021). Endocytosis of the glutamate transporter 1 is regulated by laforin and malin: implications in Lafora disease. Glia.

[26] Garcia-Cabrero AM, Sanchez-Elexpuru G, Serratosa JM, Sanchez MP (2014). Enhanced sensitivity of laforin- and malin-deficient mice to the convulsant agent pentylenetetrazole. Front Neurosci.

[27] Duran J, Gruart A, Garcia-Rocha M, Delgado-Garcia JM, Guinovart JJ (2014). Glycogen accumulation underlies neurodegeneration and autophagy impairment in Lafora disease. Hum Mol Genet.

[28] Berthier A, Paya M, Garcia-Cabrero AM, Ballester MI, Heredia M, Serratosa JM, Sanchez MP, Sanz P (2016). Pharmacological interventions to ameliorate neuropathological symptoms in a mouse model of Lafora disease. Mol Neurobiol.

[29] Molla B, Heredia M, Sanz P (2021). Modulators of neuroinflammation have a beneficial effect in a Lafora disease mouse model. Mol Neurobiol.

[30] Bissaro M, Moro S (2019). Rethinking to riluzole mechanism of action: the molecular link among protein kinase CK1delta activity, TDP-43 phosphorylation, and amyotrophic lateral sclerosis pharmacological treatment. Neural Regen Res.

[31] Kennel P, Revah F, Bohme GA, Bejuit R, Gallix P, Stutzmann JM, Imperato A, Pratt J (2000). Riluzole prolongs survival and delays muscle strength deterioration in mice with progressive motor neuronopathy (pmn). J Neurol Sci.

[32] Hunsberger HC, Weitzner DS, Rudy CC, Hickman JE, Libell EM, Speer RR, Gerhardt GA, Reed MN (2015). Riluzole rescues glutamate alterations, cognitive deficits, and tau pathology associated with P301L tau expression. J Neurochem.

[33] Hascup KN, Findley CA, Britz J, Esperant-Hilaire N, Broderick SO, Delfino K, Tischkau S, Bartke A (2021). Riluzole attenuates glutamatergic tone and cognitive decline in AbetaPP/PS1 mice. J Neurochem.

[34] Rogawski MA, Wenk GL (2003). The neuropharmacological basis for the use of memantine in the treatment of Alzheimer’s disease. CNS Drug Rev.

[35] Wenk GL, Parsons CG, Danysz W (2006). Potential role of N-methyl-D-aspartate receptors as executors of neurodegeneration resulting from diverse insults: focus on memantine. Behav Pharmacol.

[36] Repossi G, Das UN, Eynard AR (2020). Molecular basis of the beneficial actions of resveratrol. Arch Med Res.

[37] Meng T, Xiao D, Muhammed A, Deng J, Chen L, He J (2021). Anti-inflammatory action and mechanisms of resveratrol. Molecules.

[38] Capiralla H, Vingtdeux V, Zhao H, Sankowski R, Al-Abed Y, Davies P, Marambaud P (2012). Resveratrol mitigates lipopolysaccharide- and Abeta-mediated microglial inflammation by inhibiting the TLR4/NF-kappaB/STAT signaling cascade. J Neurochem.

[39] Venigalla M, Sonego S, Gyengesi E, Sharman MJ, Munch G (2016). Novel promising therapeutics against chronic neuroinflammation and neurodegeneration in Alzheimer’s disease. Neurochem Int.

[40] Garrido-Mesa N, Zarzuelo A, Galvez J (2013). Minocycline: far beyond an antibiotic. Br J Pharmacol.

[41] Moller T, Bard F, Bhattacharya A, Biber K, Campbell B, Dale E, Eder C, Gan L (2016). Critical data-based re-evaluation of minocycline as a putative specific microglia inhibitor. Glia.

[42] Li J, Sung M, Rutkove SB (2013). Electrophysiologic biomarkers for assessing disease progression and the effect of riluzole in SOD1 G93A ALS mice. PLoS ONE.

[43] Schmidt J, Schmidt T, Golla M, Lehmann L, Weber JJ, Hubener-Schmid J, Riess O (2016). In vivo assessment of riluzole as a potential therapeutic drug for spinocerebellar ataxia type 3. J Neurochem.

[44] Canistro D, Bonamassa B, Pozzetti L, Sapone A, Abdel-Rahman SZ, Biagi GL, Paolini M (2009). Alteration of xenobiotic metabolizing enzymes by resveratrol in liver and lung of CD1 mice. Food Chem Toxicol.

[45] Singleton RH, Yan HQ, Fellows-Mayle W, Dixon CE (2010). Resveratrol attenuates behavioral impairments and reduces cortical and hippocampal loss in a rat controlled cortical impact model of traumatic brain injury. J Neurotrauma.

[46] Molinaro G, Battaglia G, Riozzi B, Di Menna L, Rampello L, Bruno V, Nicoletti F (2009). Memantine treatment reduces the expression of the K(+)/Cl(-) cotransporter KCC2 in the hippocampus and cerebral cortex, and attenuates behavioural responses mediated by GABA(A) receptor activation in mice. Brain Res.

[47] Ahmadirad N, Shojaei A, Javan M, Pourgholami MH, Mirnajafi-Zadeh J (2014). Effect of minocycline on pentylenetetrazol-induced chemical kindled seizures in mice. Neurol Sci.

[48] Lalonde R, Strazielle C (2011). Brain regions and genes affecting limb-clasping responses. Brain Res Rev.

[49] Guyenet SJ, Furrer SA, Damian VM, Baughan TD, La Spada AR, Garden GA (2010) A simple composite phenotype scoring system for evaluating mouse models of cerebellar ataxia. J Vis Exp (39). 10.3791/1787 10.3791/1787 PMC3121238 20495529

[50] Seibenhener ML, Wooten MC (2015). Use of the open field maze to measure locomotor and anxiety-like behavior in mice. J Vis Exp.

[51] Walf AA, Frye CA (2007). The use of the elevated plus maze as an assay of anxiety-related behavior in rodents. Nat Protoc.

[52] Abdel Rasheed NO, El Sayed NS, El-Khatib AS (2018). Targeting central beta2 receptors ameliorates streptozotocin-induced neuroinflammation via inhibition of glycogen synthase kinase3 pathway in mice. Prog Neuropsychopharmacol Biol Psychiatry.

[53] El Sayed NS, Ghoneum MH (2020). Antia, a natural antioxidant product, attenuates cognitive dysfunction in streptozotocin-induced mouse model of sporadic alzheimer's disease by targeting the amyloidogenic, inflammatory, autophagy, and oxidative stress pathways. Oxid Med Cell Longev.

[54] RStudio_Team (2020) RStudio: Integrated development for R. RStudio, PBC, Boston, MA. https://www.rstudio.com/

[55] Cohen J (1988) Statistical power analysis for the behavioral sciences (2nd edition). Routledge, New York. 10.4324/9780203771587

[56] Kraemer HC, Morgan GA, Leech NL, Gliner JA, Vaske JJ, Harmon RJ (2003). Measures of clinical significance. J Am Acad Child Adolesc Psychiatry.

[57] Felsky D, Roostaei T, Nho K, Risacher SL, Bradshaw EM, Petyuk V, Schneider JA, Saykin A (2019). Neuropathological correlates and genetic architecture of microglial activation in elderly human brain. Nat Commun.

[58] Ahonen S, Nitschke S, Grossman TR, Kordasiewicz H, Wang P, Zhao X, Guisso DR, Kasiri S (2021). Gys1 antisense therapy rescues neuropathological bases of murine Lafora disease. Brain.

[59] Tang B, Frasinyuk MS, Chikwana VM, Mahalingan KK, Morgan CA, Segvich DM, Bondarenko SP, Mrug GP (2020). Discovery and development of small-molecule inhibitors of glycogen synthase. J Med Chem.

[60] Brewer MK, Uittenbogaard A, Austin GL, Segvich DM, DePaoli-Roach A, Roach PJ, McCarthy JJ, Simmons ZR (2019). Targeting pathogenic Lafora bodies in Lafora disease using an antibody-enzyme fusion. Cell Metab.

[61] Austin GL, Simmons ZR, Klier JE, Rondon A, Hodges BL, Shaffer R, Aziz NM, McKnight TR (2019). Central nervous system delivery and biodistribution analysis of an antibody-enzyme fusion for the treatment of Lafora disease. Mol Pharm.

[62] Israelian L, Wang P, Gabrielian S, Zhao X, Minassian BA (2020). Ketogenic diet reduces Lafora bodies in murine Lafora disease. Neurol Genet.

[63] Mohammadzadeh S, Ahangari TK, Yousefi F (2019). The effect of memantine in adult patients with attention deficit hyperactivity disorder. Hum Psychopharmacol.

[64] Dirani M, Nasreddine W, Abdulla F, Beydoun A (2014). Seizure control and improvement of neurological dysfunction in Lafora disease with perampanel. Epilepsy Behav Case Rep.

[65] Goldsmith D, Minassian BA (2016). Efficacy and tolerability of perampanel in ten patients with Lafora disease. Epilepsy Behav.

[66] Dasgupta B, Milbrandt J (2007). Resveratrol stimulates AMP kinase activity in neurons. Proc Natl Acad Sci U S A.

[67] Porro C, Cianciulli A, Calvello R, Panaro MA (2015). Reviewing the role of resveratrol as a natural modulator of microglial activities. Curr Pharm Des.

[68] Gonzalez JC, Egea J, Del Carmen GM, Fernandez-Gomez FJ, Sanchez-Prieto J, Gandia L, Garcia AG, Jordan J (2007). Neuroprotectant minocycline depresses glutamatergic neurotransmission and Ca(2+) signalling in hippocampal neurons. Eur J Neurosci.

[69] Lu Y, Yang Y, Chen W, Du N, Du Y, Gu H, Liu Q (2021). Minocycline, but not doxycycline attenuates NMDA-induced [Ca2+]i and excitotoxicity. NeuroReport.

[70] Nie H, Zhang H, Weng HR (2010). Minocycline prevents impaired glial glutamate uptake in the spinal sensory synapses of neuropathic rats. Neuroscience.

