# Supplementary material for: Pharmacological Modulation of Glutamatergic and Neuroinflammatory Pathways in a Lafora Disease Mouse Model
Source: Mol Neurobiol. 2022 Jul 14;59(10):6018–32. doi: 10.1007/s12035-022-02956-7 (PMC9463199; doi:10.1007/s12035-022-02956-7)
Supplement: Supplementary file 1 — Supplementary file1 (PDF 219 KB) [file 12035_2022_2956_MOESM1_ESM.pdf]

SUPPLEMENTARY INFORMATION

Fig. S1: Experimental design and timeline of a battery of behavioral tests. Different pre-clinical trials were designed based on both the pathway (oral or intraperitoneal injection) and the drug solvent of the administration (water, saline, or vehicle). Thus, three pre-clinical trials were performed separately and consecutively: 1) the riluzole study was performed by oral administration in drinking water; 2) the resveratrol trial by intraperitoneal administration using vehicle solution (4% ethanol, 75 mM NaCl, 2.5% PEG4000, and 2.5% Tween20); and finally, 3) the memantine and minocycline assay by intraperitoneal administration in saline solution. Treatments started in mice of 3 months of age and were maintained for two months. The cartoon is an example of intraperitoneal administration. Then mice were subjected to a battery of behavioral tests over a period of 3 weeks consisting of hindlimb clasping, open field, elevated plus maze, Y-maze spontaneous alternation, and object location memory. A description of the characteristics of each test and the sequence time in the performance is indicated. Finally, the animals were euthanized and several histopathological analyses were performed on the brain samples.

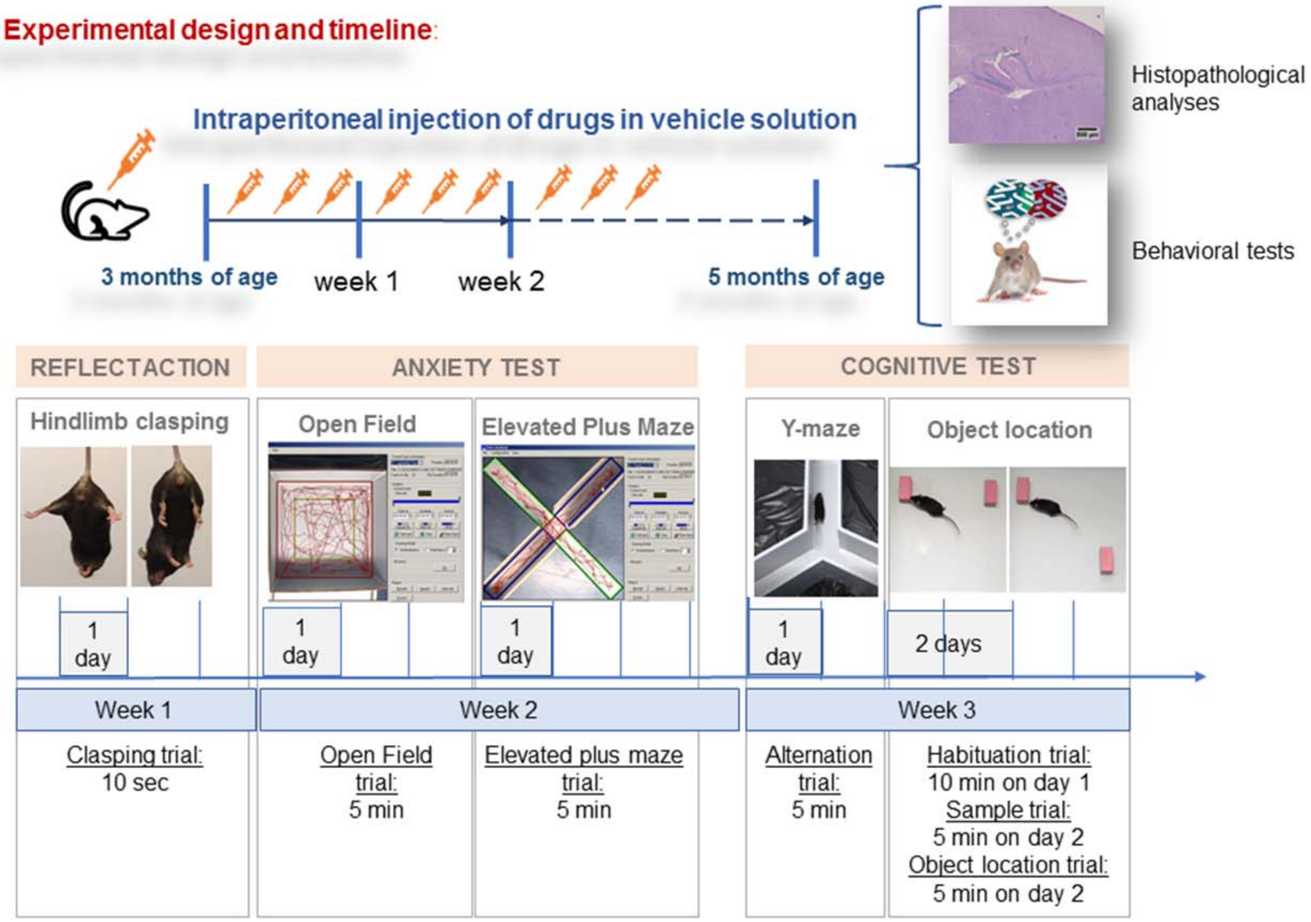

Fig. S1

Table S1: Descriptive and inferential statistical summary table of behavioral and histopathological variables analyzed throughout this study. The table details the behavioral test or histological analysis with the variable name and the statistical test used ([a] no parametric or [b] parametric or [c] parametric equal variances not assumed), the corresponding test statistic (chi-squared for non-parametric test or F test for a parametric test) and significance (p-value): *p<0.05, **p<0.01, ***p<0.001. To assess the effect size of the interventions in multiple comparisons, Cohen's delta coefficient (d) was calculated and scored as negligible (d < 0.20), small (d≥0.20), medium (d≥0.50), large (d≥0.80), and much larger (d≥1.00) size effect (see Materials and Methods). Table shows mean ± sem for each variable with the significance (p-value) and the effect size of the multiple comparisons between-groups and within-groups. In bold, significant values p<0.05 and large or much large delta coefficients.

Variable<br>Statistical test	Test statistic<br>P-value	Genotype	Control	Treatment	P value / delta	
**Openfield: % Travelled distance in center [b)]**	F = 0.376<br>p=0.544		**Water**	**Riluzole**	**within-group effects**	
		**WT**	20.61 ± 4.00	21.21 ± 2.17	p=0.9986	d = - 0.0699<br>negligible
		**KO**	27.58 ± 2.69	24.71 ± 2.34	p=0.8964	d = 0.3864<br>small
		**between-group effect**	p=0.4197	p=0.7406		
			d = - 0.7735<br>medium	d = - 0.4666<br>small		
**Openfield: % Travelled distance in center [a)]**	σ2d = 6.273<br>p=0.099		**Saline**	**Memantine**	**within-group effects**	
		**WT**	19.97 ± 2.13			
		**KO**	26.17 ± 2.49	24.36 ± 2.79	p=0.5930	d = 0.1794<br>negligible
		**between-group effect**	p=0.0730			
			d = - 0.7132<br>medium			
**Openfield: % Travelled distance in center [a)]**	σ2d = 10.14<br>**p=0.017***		**Vehicle**	**Resveratrol**	**within-group effects**	
		**WT**	16.38 ± 2.27	22.5 ± 2.31	p=0.1084	d = - 0.6892<br>medium
		**KO**	22.10 ± 2.77	14.64 ± 2.15	**p=0.0108***	**d = 0.7939<br>large**
		**between-group effect**	p=0.1661	**p=0.0053****		
			d = - 0.5954<br>small	**d = 0.9075<br>large**		
**Openfield: % Travelled distance in center [a)]**	σ2d = 6.273<br>p=0.099		**Saline**	**Minocycline**	**within-group effects**	
		**WT**	19.97 ± 2.13			
		**KO**	26.17 ± 2.49	18.01 ± 2.24	**p=0.0320***	**d = 0.9525<br>large**
		**between-group effect**	**p=0.0730**			
			d = - 0.7132<br>medium			

<table>
<tr><th>Variable
Statistical test</th><th>Test statistic P-value</th><th>Genotype</th><th>Control</th><th>Treatment</th><th colspan="2">P value / delta</th></tr>
<tr><td rowspan="5">Openfield: Entries into center (nº) [b)]</td><td rowspan="5">F = 0.376
p=0.544</td><td></td><td>Water</td><td>Riluzole</td><td colspan="2">within-group effects</td></tr>
<tr><td>WT</td><td>15.42 ± 3.21</td><td>12.5 ± 1.99</td><td>p=0.8944</td><td>d = 0.3960 small</td></tr>
<tr><td>KO</td><td>22.83 ± 4.44</td><td>18.68 ± 2.73</td><td>p=0.7773</td><td>d = 0.4274 small</td></tr>
<tr><td rowspan="2">between-group effect</td><td>p=0.4219</td><td>p=0.3475</td><td colspan="2" rowspan="2"></td></tr>
<tr><td>d = - 0.7657 medium</td><td>d = - 0.7784 medium</td></tr>
<tr><td rowspan="5">Openfield: Entries into center (nº) [a)]</td><td rowspan="5">σ2d = 9.966
p=0.018*</td><td></td><td>Saline</td><td>Memantine</td><td colspan="2">within-group effects</td></tr>
<tr><td>WT</td><td>16.92 ± 2.19</td><td></td><td></td><td></td></tr>
<tr><td>KO</td><td>21.92 ± 2.53</td><td>17.46 ± 2.92</td><td>p=0.1669</td><td>d = 0.4295 small</td></tr>
<tr><td rowspan="2">between-group effect</td><td>p=0.2386</td><td></td><td colspan="2" rowspan="2"></td></tr>
<tr><td>d = - 0.5642 medium</td><td></td></tr>
<tr><td rowspan="5">Openfield: Entries into center (nº) [a)]</td><td rowspan="5">σ2d = 4.028
p=0.258</td><td></td><td>Vehicle</td><td>Resveratrol</td><td colspan="2">within-group effects</td></tr>
<tr><td>WT</td><td>10.86 ± 2.30</td><td>15.93 ± 2.24</td><td>p=0.0570</td><td>d = - 0.5749 medium</td></tr>
<tr><td>KO</td><td>13.42 ± 1.51</td><td>12.00 ± 1.63</td><td>p=0.5310</td><td>d = 0.2369 small</td></tr>
<tr><td rowspan="2">between-group effect</td><td>p=0.1710</td><td>p=0.2530</td><td colspan="2" rowspan="2"></td></tr>
<tr><td>d = - 0.3402 small</td><td>d = 0.5163 medium</td></tr>
<tr><td rowspan="5">Openfield: Entries into center (nº) [a)]</td><td rowspan="5">σ2d = 9.966
p=0.018*</td><td></td><td>Saline</td><td>Minocycline</td><td colspan="2">within-group effects</td></tr>
<tr><td>WT</td><td>16.92 ± 2.19</td><td></td><td></td><td></td></tr>
<tr><td>KO</td><td>21.92 ± 2.53</td><td>9.63 ± 1.47</td><td>p=0.0017**</td><td>d = 1.5724 much large</td></tr>
<tr><td rowspan="2">between-group effect</td><td>p=0.2386</td><td></td><td colspan="2" rowspan="2"></td></tr>
<tr><td>d = - 0.5642 medium</td><td></td></tr>
</table>

Variable<br>Statistical test	Test statistic<br>P-value	Genotype	Control	Treatment	P value / delta	
**Elevated plus maze: %Permanence time in open arms** [a)]	σ2d = 4.658<br>p=0.198		**Water**	**Riluzole**	**within-group effects**	
		**WT**	9.18 ± 2.61	18.47 ± 4.99	p=0.1830	d = - 0.7139<br>medium
		**KO**	23.88 ± 5.60	20.03 ± 6.46	p=0.4010	d = 0.2002<br>small
		**between-group effect**	**p=0.0340***	p=0.8200		
			**d = - 1.3906<br>much large**	d = - 0.0825<br>negligible		
**Elevated plus maze: %Permanence time in open arms** [a)]	σ2d = 5.675<br>p=0.339		**Saline**	**Memantine**	**within-group effects**	
		**WT**	18.26 ± 3.48			
		**KO**	20.56 ± 3.77	18.53 ± 3.51	p=0.8500	d = 0.1409<br>negligible
		**between-group effect**	p=0.7500			
			d = - 0.1605<br>negligible			
**Elevated plus maze: %Permanence time in open arms** [b)]	F = 2.766<br>p=0.101		**Vehicle**	**Resveratrol**	**within-group effects**	
		**WT**	19.30 ± 3.02	13.28 ± 1.83	p=0.3772	d = 0.6216<br>medium
		**KO**	14.79 ± 2.39	17.60 ± 3.12	p=0.8795	d = - 0.2629<br>small
		**between-group effect**	p=0.6352	p=0.6533		
			d = 0.4305<br>small	d = - 0.4360<br>small		
**Elevated plus maze: %Permanence time in open arms** [a)]	σ2d = 5.675<br>p=0.339		**Saline**	**Minocycline**	**within-group effects**	
		**WT**	18.26 ± 3.48			
		**KO**	20.56 ± 3.77	26.00 ± 5.27	p=0.8500	d = - 0.3063<br>small
		**between-group effect**	p=0.7500			
			d = - 0.1605<br>negligible			

**Variable** Statistical test	**Test statistic P-value**	**Genotype**	**Control**	**Treatment**	**P value / delta**	
**Elevated plus maze: Entries into arms [b)]**	F = 0.744 p=0.394		**Water**	**Riluzole**	**within-group effects**	
		**WT**	32.71 ± 5.72	33.54 ± 5.75	p=0.9995	d = - 0.0469 negligible
		**KO**	41.00 ± 6.40	32.63 ± 2.88	p=1.0000	d = 0.6991 medium
		**between-group effect**	p=0.7621	p=0.9995		
			d = - 0.5383 medium	d = 0.0601 negligible		
**Elevated plus maze: Entries into arms [a)]**	σ2d = 4.329 p=0.228		**Saline**	**Memantine**	**within-group effects**	
		**WT**	32.93 ± 4.23			
		**KO**	38.53 ± 4.38	38.75 ± 6.04	p=0.6600	d = - 0.0103 negligible
		**between-group effect**	p=0.3100			
			d = - 0.3300 small			
**Elevated plus maze: Entries into arms [b)]**	F = 0.229 p=0.633		**Vehicle**	**Resveratrol**	**within-group effects**	
		**WT**	31.73 ± 3.41	34.4 ± 3.51	p=0.9601	d = - 0.1987 negligible
		**KO**	34.35 ± 3.15	33.33 ± 4.97	p=0.9976	d = 0.0635 negligible
		**between-group effect**	p=0.9637	p=0.9972		
			d = - 0.2090 small	d = 0.0639 negligible		
**Elevated plus maze: Entries into arms [a)]**	σ2d = 4.329 p=0.228		**Saline**	**Minocycline**	**within-group effects**	
		**WT**	32.93 ± 4.23			
		**KO**	38.53 ± 4.38	25.33 ± 3.33	**p=0.0500**	**d = 0.8753 large**
		**between-group effect**	p=0.3100			
			d = - 0.3300 small			

Variable<br>Statistical test	Test statistic<br>P-value	Genotype	Control	Treatment	P value / delta	
**Y-maze: % Alternations [b)]**	F = 0.597<br>p=0.445		**Water**	**Riluzole**	**within-group effects**	
		**WT**	70.66 ± 6.42	62.01 ± 4.97	p=0.5683	d = 0.5493<br>medium
		**KO**	58.43 ± 2.51	56.98 ± 3.20	p=0.9960	d = 0.1550<br>negligible
		**between-group effect**	p=0.3689	p=0.8069		
			**d = 1.0232<br>much large**	d = 0.3732<br>small		
**Y-maze: % Alternations [a)]**	σ2d = 1.050<br>p=0.789		**Saline**	**Memantine**	**within-group effects**	
		**WT**	55.89 ± 4.64			
		**KO**	49.22 ± 3.81	54.61 ± 5.57	p=0.7200	d = - 0.2832<br>small
		**between-group effect**	p=0.3100			
			d = 0.4055<br>small			
**Y-maze: % Alternations [b)]**	F = 0.187<br>p=0.666		**Vehicle**	**Resveratrol**	**within-group effects**	
		**WT**	60.24 ± 3.84	52.37 ± 4.09	p=0.5813	d = 0.5208<br>medium
		**KO**	60.23 ± 5.45	48.55 ± 4.05	p=0.2562	d = 0.6492<br>medium
		**between-group effect**	p=1.0000	p=0.9286		
			d = 0.0008<br>negligible	d = 0.2504<br>small		
**Y-maze: % Alternations [a)]**	σ2d = 1.050<br>p=0.789		**Saline**	**Minocycline**	**within-group effects**	
		**WT**	55.89 ± 4.64			
		**KO**	49.22 ± 3.81	54.65 ± 7.35	p=0.7100	d = - 0.2583<br>small
		**between-group effect**	p=0.3100			
			d = 0.4055<br>small			

Variable<br>Statistical test	Test statistic<br>P-value	Genotype	Control	Treatment	P value / delta	
Y-maze: % Incomplete alternations [b)]	F = 0.944<br>p=0.339		**Water**	**Riluzole**	**within-group effects**	
		**WT**	33.14 ± 7.03	39.48 ± 8.51	p=0.9497	d = - 0.2650<br>small
		**KO**	53.54 ± 9.10	43.74 ± 6.56	p=0.8342	d = 0.4459<br>small
		**between-group effect**	p=0.4271	p=0.9736		
			**d = - 1.0233<br>much large**	d = - 0.1751<br>negligible		
Y-maze: % Incomplete alternations [a)]	σ2d = 6.958<br>p=0.073		**Saline**	**Memantine**	**within-group effects**	
		**WT**	15.03 ± 5.90			
		**KO**	40.38 ± 7.40	19.11 ± 6.18	**p=0.0350***	d = 0.7955<br>medium
		**between-group effect**	**p=0.0170***			
			**d = - 0.9767<br>large**			
Y-maze: % Incomplete alternations [b)]	F = 0.264<br>p=0.609		**Vehicle**	**Resveratrol**	**within-group effects**	
		**WT**	37.83 ± 6.75	27.11 ± 7.27	p=0.7410	d = 0.4016<br>small
		**KO**	35.04 ± 8.90	32.07 ± 7.06	p=0.9924	d = 0.0988<br>negligible
		**between-group effect**	p=0.9934	p=0.9670		
			d = 0.0934<br>negligible	d = - 0.1846<br>negligible		
Y-maze: % Incomplete alternations [a)]	σ2d = 6.958<br>p=0.073		**Saline**	**Minocycline**	**within-group effects**	
		**WT**	15.03 ± 5.90			
		**KO**	40.38 ± 7.40	20.46 ± 6.06	p=0.0780	d = 0.7955<br>medium
		**between-group effect**	**p=0.0170***			
			**d = - 0.9767<br>large**			

Variable<br>Statistical test	Test statistic<br>P-value	Genotype	Control	Treatment	P value / delta	
			**Water**	**Riluzole**	**within-group effects**	
		**WT**	-30.26 ± 11.38	-14.88 ± 11.97	p=0.8405	d = - 0.4222 small
**OLM: DI** [b)]	F = 3.197<br>p=0.083	**KO**	19.78 ± 8.34	-12.95 ± 14.26	p=0.3508	**d = 0.8106 large**
		**between-group effect**	p=0.1100	p=0.9994		
			**d = - 1.9123 much large**	d = - 0.0442 negligible		
			**Saline**	**Memantine**	**within-group effects**	
		**WT**	-10.79 ± 7.68			
**OLM: DI** [b)]	F = 0.659<br>p=0.580	**KO**	-2.93 ± 7.74	-14.36 ± 5.97	p=0.6742	d = 0.4265 small
		**between-group effect**	p=0.8596			
			d = - 0.2585 small			
			**Vehicle**	**Resveratrol**	**within-group effects**	
		**WT**	-23.74 ± 7.09	-15.17 ± 6.12	p=0.2200	d = - 0.3334 small
**OLM: DI** [a)]	σ2d = 2.632<br>p=0.451	**KO**	-13.77 ± 9.56	-7.31 ± 10.02	p=0.5700	d = - 0.1727 negligible
		**between-group effect**	p=0.3500	p=0.7500		
			d = - 0.3138 small	d = - 0.2444 small		
			**Saline**	**Minocycline**	**within-group effects**	
		**WT**	-10.79 ± 7.68			
**OLM: DI** [b)]	F = 0.659<br>p=0.580	**KO**	-2.93 ± 7.74	-2.59 ± 6.97	p=0.9999	d = - 0.0122 negligible
		**between-group effect**	p=0.8596			
			d = - 0.2585 small			

Variable<br>Statistical test	Test statistic<br>P-value	Genotype	Control	Treatment	P value / delta	
**OLM: Activity time (s)** [b)]	F = 0.0001<br>p=0.991		**Water**	**Riluzole**	**within-group effects**	
		**WT**	107.85 ± 21.21	138.45 ± 14.17	p=0.4728	d = - 0.6042<br>medium
		**KO**	102 ± 9.80	132.90 ± 11.40	p=0.5056	**d = - 0.9131<br>large**
		**between-group effect**	p=0.9949	p= 0.9904		
			d = 0.1315<br>negligible	d = 0.1299<br>negligible		
**OLM: Activity time (s)** [a)]	σ2d = 6.619<br>p=0.085		**Saline**	**Memantine**	**within-group effects**	
		**WT**	157 ± 12.22			
		**KO**	196.86 ± 14.10	145.26 ± 15.73	**p=0.0160***	**d = 0.8917<br>large**
		**between-group effect**	**p=0.0480***			
			**d = - 0.7706<br>medium**			
**OLM: Activity time (s)** [a)]	σ2d = 1.832<br>p=0.608		**Vehicle**	**Resveratrol**	**within-group effects**	
		**WT**	87.66 ± 9.21	73.33 ± 7.70	p=0.2000	d = 0.4356<br>small
		**KO**	80.35 ± 10.78	81.53 ± 11.08	p=0.9200	d = - 0.0282<br>negligible
		**between-group effect**	p=0.3900	p=0.7600		
			d = 0.1923<br>negligible	d = - 0.2218<br>small		
**OLM: Activity time (s)** [a)]	σ2d = 6.619<br>p=0.085		**Saline**	**Minocycline**	**within-group effects**	
		**WT**	157 ± 12.22			
		**KO**	196.86 ± 14.10	168.00 ± 12.15	p=0.331	d = 0.5725<br>medium
		**between-group effect**	**p=0.0480***			
			**d = - 0.7706<br>medium**			

Variable Statistical test	Test statistic P-value	Genotype	Control	Treatment	P value / delta	
PAS: % PGs number vs KO[c)]	F =20.68 p=0.0008***		Water	Riluzole	within-group effects	
		WT	1.18 ± 0.77	0.78 ± 0.21	p=0.9500	d = 0.4103 small
		KO	100.00 ± 17.31	60.23 ± 9.26	p=0.2600	d = 1.1345 much large
		between-group effect	p=0.0100**	p=0.0010**		
			d = - 2.7564 much large	d = - 2.1199 much large		
PAS: % PGs number vs KO[a)]	σ2d = 8.714 p=0.033*		Saline	Memantine	within-group effects	
		WT	3.54 ± 0.26			
		KO	100.00 ± 4.71	84.76 ± 8.79	p=0.2642	d = 0.8801 large
		between-group effect	p=0.0056**			
			d = - 9.8801 much large			
PAS: % PGs number vs KO[a)]	σ2d = 14.44 p=0.002**		Vehicle	Resveratrol	within-group effects	
		WT	12.22 ± 2.95	124.65 ± 3.15	p=0.9021	d = - 0.4583 small
		KO	100.00 ± 7.26	98.34 ± 8.58	p=0.8640	d = 0.0548 negligible
		between-group effect	p=0.0051**	p=0.0101*		
			d = - 3.6061 much large	d = - 2.6847 much large		
PAS: % PGs number vs KO[a)]	σ2d = 8.714 p=0.033*		Saline	Minocycline	within-group effects	
		WT	3.54 ± 0.26			
		KO	100.00 ± 4.71	98.03 ± 4.51	p=0.7801	d = 0.1738 negligible
		between-group effect	p=0.0056**			
			d = - 9.8801 much large			

Variable<br>Statistical test	Test statistic<br>P-value	Genotype	Control	Treatment	P value / delta	
Astrogliosis: % GFAP area vs KO[a)]	σ2d = 6.390<br>p=0.094		**Water**	**Riluzole**	**within-group effects**	
		**WT**	78.47 ± 15.55	51.77 ± 8.31	p=0.1859	**d = 0.8091 large**
		**KO**	100.00 ± 14.22	93.44 ± 13.29	p=0.6434	d = 0.1589 negligible
		**between-group effect**	p=0.3161	**p=0.0331***		
			d = - 0.5604 medium	**d = - 1.1148 much large**		
Astrogliosis: % GFAP area vs KO[a)]	σ2d = 9.553<br>**p=0.022***		**Saline**	**Memantine**	**within-group effects**	
		**WT**	38.98 ± 7.05			
		**KO**	100.00 ± 9.57	89.10 ± 14.95	p=0.3691	d = 0.3541 small
		**between-group effect**	**p=0.0062****			
			**d = - 2.9610 much large**			
Astrogliosis: % GFAP area vs KO [b)]	F = 0.324<br>p=0.576		**Vehicle**	**Resveratrol**	**within-group effects**	
		**WT**	57.13 ± 9.65	54.95 ± 4.67	p=0.9980	d = 0.1290 negligible
		**KO**	100.00 ± 10.25	107.88 ± 9.61	p=0.9207	d = - 0.3545 small
		**between-group effect**	**p=0.0163***	**p=0.0031****		
			**d = - 1.6245 much large**	**d = - 3.1300 much large**		
Astrogliosis: % GFAP area vs KO[a)]	σ2d = 9.553<br>**p=0.022***		**Saline**	**Minocycline**	**within-group effects**	
		**WT**	38.98 ± 7.05			
		**KO**	100.00 ± 9.57	90.67 ± 7.17	p=0.9025	d = 0.4500 small
		**between-group effect**	**p=0.0062****			
			**d = - 2.9610 much large**			

Variable<br>Statistical test	Test statistic<br>P-value	Genotype	Control	Treatment	P value / delta	
**Microgliosis: % Iba1+ cells vs KO[a)]**	σ2d = 12.54<br>**p=0.005****		**Water**	**Riluzole**	**within-group effects**	
		**WT**	75.64 ± 4.49	55.67 ± 4.26	**p=0.0200***	**d = 1.7217 much large**
		**KO**	100.00 ± 13.71	71.75 ± 4.82	p=0.0900	**d = 1.2078 much large**
		**between-group effect**	p=0.2123	**p=0.0250***		
			**d = - 1.0024 much large**	**d = - 1.1157 much large**		
**Microgliosis: % Iba1+ cells vs KO[b)]**	F = 1.051<br>p=0.391		**Saline**	**Memantine**	**within-group effects**	
		**WT**	77.18 ± 8.13			
		**KO**	100.00 ± 11.48	105.42 ± 13.91	p=0.9881	d = - 0.1736 negligible
		**between-group effect**	p=0.5432			
			**d = - 0.9355 large**			
**Microgliosis: % Iba1+ cells vs KO[b)]**	F = 1.838<br>p=0.193		**Vehicle**	**Resveratrol**	**within-group effects**	
		**WT**	78.82 ± 7.61	92.88 ± 7.61	p=0.4968	**d = -0.8259 large**
		**KO**	100.00 ± 4.54	95.27 ± 7.44	p=0.9619	d = 0.3426 small
		**between-group effect**	p=0.1763	p=0.9946		
			**d = - 1.5103 much large**	d = - 0.1421 negligible		
**Microgliosis: % Iba1+ cells vs KO[b)]**	F = 1.051<br>p=0.391		**Saline**	**Minocycline**	**within-group effects**	
		**WT**	77.18 ± 8.13			
		**KO**	100.00 ± 11.48	92.96 ± 13.37	p=0.9750	d = 0.2302 small
		**between-group effect**	p=0.5432			
			**d = - 0.9355 large**			

Table S2: Contingency tables and inferential statistical summary of hindlimb clasping test. The summary table details the contingency tables of the relative frequency of the hindlimb clasping score (from absent to severe) for each group of treatment. Pearson's Chi-square test [a)] was used to estimate the chi-squared statistic (σ2d) and the p-value (p) for multiple and paired comparisons between-groups and within-groups. Fischer’s exact test [b)] was used when sample sizes were zero. Significance (p-value): ***p<0.001, ****p<0.0001.

Test statistic P-value	Genotype	Control				Treatment				P value / chi square	
		**Water**				**Riluzole**				**within-group effects**	
	**WT**	Ab	Mi	Mo	Se	Ab	Mi	Mo	Se	p=5.335e-06**** [b)]	σ2d = NA [b)]
		85.71	14.29	0.00	0.00	72.73	9.09	18.18	0.00		
σ2d = 131.9 p=2.20e-16****	**KO**	Ab	Mi	Mo	Se	Ab	Mi	Mo	Se	p=2.143e-10****	σ2d = 47.99
		66.67	16.67	16.67	0.00	27.27	9.09	54.55	9.09		
	**between-group effect**	p=7.551e-06**** [b)]				p=2.205e-10****					
		σ2d = NA [b)]				σ2d = 47.93					
		**Saline**				**Memantine**				**within-group effects**	
	**WT**	Ab	Mi	Mo	Se						
		68.75	33.33	43.75	66.66						
σ2d = 84.02 p=2.55e-14****	**KO**	Ab	Mi	Mo	Se	Ab	Mi	Mo	Se	p=0.0007***	σ2d = 16.938
		33.33	33.33	40.00	6.66	43.75	12.50	43.75	0.00		
	**between-group effect**	p=9.97e-08****									
		σ2d = 35.410									
		**Vehicle**				**Resveratrol**				**within-group effects**	
	**WT**	Ab	Mi	Mo	Se	Ab	Mi	Mo	Se	p= 0.4208[b)]	σ2d = NA [b)]
		80.00	6.66	13.33	0.00	73.33	6.66	20.00	0.00		
σ2d = 84.93 p=1.67e-14****	**KO**	Ab	Mi	Mo	Se	Ab	Mi	Mo	Se	p=1.634e-07****	σ2d = 34.397
		35.71	28.57	21.42	14.28	73.33	6.66	6.66	13.33		
	**between-group effect**	p=3.94e-10****				p=0.0001***					
		σ2d = 46.742				σ2d = 20.005					
		**Saline**				**Minocycline**				**within-group effects**	
	**WT**	Ab	Mi	Mo	Se						
		68.75	33.33	43.75	66.66						
σ2d = 84.02 p=2.55e-14****	**KO**	Ab	Mi	Mo	Se	Ab	Mi	Mo	Se	p=4.731e-09****	σ2d = 41.663
		33.33	33.33	40.00	6.66	66.66	26.66	6.66	0.00		
	**between-group effect**	p=9.97e-08****									
		σ2d = 35.410									
